# Impact of linkage level on inferences from big data analyses in health and medical research: an empirical study

**DOI:** 10.1186/s12911-024-02586-0

**Published:** 2024-07-09

**Authors:** Bora Lee, Young-Kyun Lee, Sung Han Kim, HyunJin Oh, Sungho Won, Suk-Yong Jang, Ye Jin Jeon, Bit-Na Yoo, Jean-Kyung Bak

**Affiliations:** 1 Institute of Health & Environment, Seoul National University, Seoul, Republic of Korea; 2 Department of Orthopedic Surgery, Seoul National University College of Medicine and Seoul National University Bundang Hospital, Seongnam-si, Republic of Korea; 3 Department of Urology, Urologic Cancer Center, Research Institute and Hospital of National Cancer Center, Goyang-si, Republic of Korea; 4 Division of Gastroenterology, Department of Internal Medicine, Center for Cancer Prevention and Detection of National Cancer Center, Goyang-si, Republic of Korea; 5 Department of Public Health Sciences, Graduate School of Public Health, Seoul National University, Seoul, Republic of Korea; 6 Interdisciplinary Program for Bioinformatics, College of Natural Science, Seoul National University, Seoul, Republic of Korea; 7 Department of Healthcare Management, Graduate School of Public Health, Yonsei University, Seoul, Republic of Korea; 8 Department of Public Health, Graduate School, Yonsei University, Seoul, Republic of Korea; 9 National Evidence-based Healthcare Collaborating Agency (NECA), 3-5F 400, Neungdong-ro, Gwangin-gu, Seoul, 04933 Republic of Korea

**Keywords:** Directly identifiable information, Indirectly identifiable information, Linkage levels, Accuracy

## Abstract

**Background:**

Linkage errors that occur according to linkage levels can adversely affect the accuracy and reliability of analysis results. This study aimed to identify the differences in results according to personally identifiable information linkage level, sample size, and analysis methods through empirical analysis.

**Methods:**

The difference between the results of linkage in directly identifiable information (DII) and indirectly identifiable information (III) linkage levels was set as III linkage based on name, date of birth, and sex and DII linkage based on resident registration number. The datasets linked at each level were named as database_III_ (DB_III_) and database_DII_ (DB_DII_), respectively. Considering the analysis results of the DII-linked dataset as the gold standard, descriptive statistics, group comparison, incidence estimation, treatment effect, and moderation effect analysis results were assessed.

**Results:**

The linkage rates for DB_DII_ and DB_III_ were 71.1% and 99.7%, respectively. Regarding descriptive statistics and group comparison analysis, the difference in effect in most cases was “none” to “very little.” With respect to cervical cancer that had a relatively small sample size, analysis of DB_III_ resulted in an underestimation of the incidence in the control group and an overestimation of the incidence in the treatment group (hazard ratio [HR] = 2.62 [95% confidence interval (CI): 1.63–4.23] in DB_III_ vs. 1.80 [95% CI: 1.18–2.73] in DB_DII_). Regarding prostate cancer, there was a conflicting tendency with the treatment effect being over or underestimated according to the surveillance, epidemiology, and end results summary staging (HR = 2.27 [95% CI: 1.91–2.70] in DB_III_ vs. 1.92 [95% CI: 1.70–2.17] in DB_DII_ for the localized stage; HR = 1.80 [95% CI: 1.37–2.36] in DB_III_ vs. 2.05 [95% CI: 1.67–2.52] in DB_DII_ for the regional stage).

**Conclusions:**

To prevent distortion of the analyses results in health and medical research, it is important to check that the patient population and sample size by each factor of interest (FOI) are sufficient when different data are linked using DB_DII_. In cases involving a rare disease or with a small sample size for FOI, there is a high likelihood that a DII linkage is unavoidable.

**Supplementary Information:**

The online version contains supplementary material available at 10.1186/s12911-024-02586-0.

## Background

Over the past several years, the health and medical fields have achieved breakthroughs in evidence-based personalized medical services, including medical care, service improvement, and treatment innovations [1]. Such achievements can be attributed to the continued efforts to research and develop the medical, academic, and industrial communities to collect a vast amount of data, including health examinations, electronic medical records, and genomic data, through various routes, and explore various ways of utilizing such data [2].

Starting with data collected from public agencies, each country has performed intra- or inter-agency data linkages and integration to maximize the value of big data [3, 4]. Prime examples of health and medical linked data include clinical research using linked bespoke studies and electronic health records (CALIBER) of the UK, Cancer Registry Data (Zentrum für Krebsregisterdaten, ZfKD) of Germany, National Health Data System of France, and National Center for Health Statistics – Housing and Urban Development of the US [5–9]. In 2018, the Ministry of Health and Welfare (MoHW) of Korea launched a pilot big data project to support health and medical research using linked data consisting of name, sex, and date of birth (DOB) information from four public agencies (National Health Insurance Service [NHIS], Health Insurance Review and Assessment Service [HIRA], Korea Disease Control and Prevention Agency [KDCA], and National Cancer Center [NCC]). Accordingly, various studies, including mortality trends and mortality prediction among patients with lung cancer, are being conducted [10, 11].

Inter-agency data integration in each country takes place on a limited basis within the scope permitted by law. Moreover, linkage based on non-personally identifiable information without any unique identifiers, such as resident registration number (RRN), for reasons such as the protection of DII, could lead to two types of errors: false matches and missed matches. A false match refers to the records of two different individuals being falsely linked to each other, while a missed match refers to information being omitted because the records of the same individuals are not linked to each other. Such errors can affect the bias and precision of analysis results [12]. False matches are known to cause biases in the estimates and weaken the associations between variables in different datasets even when the error rate is < 1%, while missed matches weaken the statistical power by reducing the sample size, causing underestimation [12–15].

To generate big data-based evidence in the health and medical fields, the accuracy and reliability of study results are vital. Therefore, studies on the effects of linkage errors in research findings are essential. However, such studies that have very low linkage errors have been conducted sporadically in the UK, US, and Australia, whereas studies in Brazil and Tanzania that have very high linkage errors have been conducted on very limited topics [16–19]. Such studies are insufficient to present comprehensive guidelines for research findings according to linkage accuracy and reliability.

Datasets were established for each of the five topics in databases linked using information, such as name, DOB, and sex, based on the Health Care Big Data Platform pilot project launched by the MoHW in 2018. After conducting analyses by level, including descriptive statistics, group comparison, and modeling, the results were compared with those of datasets linked to the RRN, unique identifier information, for multidimensional investigation of the effects of errors according to linkage level on inferences from health and medical research based on dataset scale and analysis methods.

## Methods

### Data source

Through the Health Care Big Data Platform pilot project launched by the MoHW in 2018, researchers have conducted various studies linking data from four public agencies (NHIS, HIRA, KDCA, and NCC). We linked NHIS claims data and NCC cancer registry data to analyze osteoporotic fractures according to treatment for five different types of cancer.

In South Korea, the Personal Information Protection Act (PIPA) governs the processing and protection of all personal information [20]. NHIS strictly adheres to the PIPA by obtaining explicit consent before processing personal information, implementing robust security measures, and promptly notifying relevant authorities and affected individuals in the event of a data breach. Therefore, every stage of this study utilizing NHIS claim data was conducted with the utmost effort to protect all personal information included in the data in accordance with the PIPA [21].

The NHIS system in South Korea requires mandatory enrollment of all citizens and determines insurance premiums based on various eligibility criteria, such as income level, and subsidizes a portion of the fees for services received from care institutions. Therefore, all citizens with RRN pay an insurance contribution to the NHIS every month, which is proportional to their income and assets [22]. The claims data of the NHIS are population-based, real-world data, which reduce selection bias and are consistently updated in a validation system [23]. Accessing claims data from the NHIS is possible only through the analysis centers after receiving permission from the National Health Insurance Data Provision Review Committee, which plays a crucial role in making decisions regarding the use and disclosure of the claims data considering issues such as the unauthorized use and protection of sensitive information during its deliberations [24]. As in other countries, the claims data in South Korea are actively assessed in terms of quality. Although there are data limitations such as difficulty in determining causal relationships for diseases, the quality of the claims data from the NHIS is being improved consistently by the deployment of specialized personnel and increasing resources [23, 25]. Accordingly, the Korea National Health Insurance Database manages data on qualification and insurance premiums, care institution usage, and examination results of all South Korean citizens from birth to death. The eligibility and insurance premium database includes data regarding DOB, sex, area of residence, insurance type, and premium quantile of each subscriber. The care detail database includes general information, such as care institution, the start date of care, number of days in care, and disease name; treatment information such as treatment and surgery in care institutions and inpatient prescriptions; and disease information, including sub-diagnosis and outpatient prescriptions. The health screening database includes health inquiry and examination data, such as actual measured data from each screening, lifestyle, family history, and disease history [26].

The Korea Central Cancer Registry (KCCR), which was established to monitor cancer incidence and manage patients with cancer at the national level, consists mainly of cancer patient data at the local level registered at training hospitals or higher-level hospitals between 1988 and 2003. It was subsequently expanded for the continuous collection of data at the national level. Among individuals who had filed an insurance claim for cancer, those with no prior diagnosis of cancer in the past three years, having a history of hospitalization for cancer in the applicable year, and the total care expense exceeding a certain amount were defined and registered as new patients with cancer. The information collected at the time of registration includes patient information and information on cancer type [27].

### Data linkage

The data were linked largely using two methods. The first method was indirectly identifiable information linkage based on name, DOB, and sex, and the other was directly identifiable information linkage based on RRN, a unique identifier (named DB_III_ and DB_DII_, respectively). The Korea Internet & Security Agency, a government-designated composite key management agency, generates composite keys based on the directly identifiable information supplied by each agency, and the keys are sent to each agency. DB_III_ and DB_DII_ are generated by combining datasets between agencies based on such keys, and they include false and missed matches. DB_III_ and DB_DII_ were generated using the same process [28].

### Research topic and participants

Bone loss among patients with cancer is associated with osteoporotic fractures caused by cancer-specific therapies, such as androgen deprivation therapy for prostate cancer and aromatase inhibitors (AIs) for breast cancer [29, 30]. Especially, one-year mortality of older adult patients with osteosarcopenic hip fractures is higher than that for patients without osteosarcopenia [31], and the five-year survival rate of osteoporotic fractures was 45.8% in South Korea [32]. Additionally, patients with breast, prostate, thyroid, cervical, or gastric cancer have a higher incidence of osteoporotic fractures than normal participants according to the NCC registry [33–35]. We evaluated the risk of fractures among Korean patients with thyroid cancer (TC), gastric cancer (GC), breast cancer (BC), prostate cancer (PC), and cervical cancer (CC). To develop an appropriate linkage method in the Health Care Big Data Platform launched by the MoHW in 2018, we conducted diverse medical and clinical studies by linking data from four public agencies (NHIS, HIRA, KDCA, and NCC). For a multidimensional comparison of the accuracy of the analysis results and linkage rate at the DII linkage level according to the dataset scale and analysis methods, five subtopics were set according to treatment groups for each type of cancer. This topic was chosen because there have been discussions about cancer-related osteoporosis and fracture, and we determined the effect of cancer as a risk factor for osteoporotic fracture in the South Korean population.

1. Incidence of osteoporotic fractures according to postoperative vitamin D use in patients with TC.
2. Incidence of osteoporotic fractures according to surgery type (total gastrectomy, subtotal gastrectomy, and endoscopic submucosal dissection (ESD)/endoscopic mucosal resection (EMR) in patients with GC.
3. Incidence of osteoporotic fractures according to the type of anti-hormone therapy (HT) prescribed to patients with BC.
4. Incidence of osteoporotic fractures according to androgen deprivation therapy (ADT) in patients with PC.
5. Incidence of osteoporotic fractures according to radiation therapy (RT) in patients with CC.

The dataset for each topic is established in two steps. First, among the new cancer cases between 2008 and 2016 available from the KCCR, patients with no history of death among adults with no cancer-related diagnosis in 2007 were defined as the initial cancer patient population. Subsequently, different inclusion and exclusion criteria that met the research topics for the characteristics of different cancers were set to establish the final dataset. Further details are provided in the additional applicable criteria file (Supplementary Table 1).

### Variable

The index date in this study was defined as the date when the treatment was administered for each cancer type. Osteoporotic fracture, which is the outcome variable, was defined as the first incidence of fracture among hip, vertebral, distal radius, and proximal humerus fractures in cancer cases, except for CC. For CC, pelvic insufficiency fracture was defined as the outcome variable. Patients with no fracture or discontinuation of follow-up owing to death or immigration were censored.

The baseline characteristics used in the analysis were age, age group (10 years), sex, area of residence, insurance type, premium quantile, and the Charlson Comorbidity Index (CCI). The age at the time of index dating was applied for age. The area of residence was divided into urban areas for 17 city/district units (Seoul, Gyeonggi-do, and metropolitan cities) and rural areas for all other areas. The types of insurance were divided into employment insurance and dependents, local insurance and dependents, and medical aid and dependents. The premium quantile was divided into 11 levels with 1–20 percentile for two levels each and medical aid as level 0. This information was considered to indicate the income level of the subscriber. CCI was defined using the criteria proposed by Quan et al. [36] based on the name of the disease diagnosed within one year prior to the initial cancer diagnosis. Depending on the number of comorbidities, the CCI was divided into 0–1, 2, 3, and ≥ 4 [36]. Further details are provided in the additional applicable information file (Supplementary Table 2).

Clinical characteristics included SEER summary staging, use of osteoporosis drugs, bone mineral density (BMD) test, and RT. For SEER summary staging, variables classified as localized, regional, primary, or unidentified based on the registration information at the time of initial diagnosis were utilized. The use of osteoporosis drugs, BMD tests, and RT was defined based on applicable prescription/fee codes within one year of the cancer diagnosis. Further details regarding the codes used for the operational definitions of the treatment, outcomes, and clinical variables are provided in the additional applicable information file (Supplementary Table 3).

### Statistical analyses

Continuous variables are expressed as numbers, mean ± standard deviation, and median (minimum and maximum) values, while categorical variables are expressed as frequencies and percentages. For continuous variables, a two-sample t-test or Mann–Whitney U test was performed to compare the two groups according to whether the normality assumption was satisfied. For the comparison of three or more groups, a one-way analysis of variance or the Kruskal-Wallis test was performed. Categorical variables were examined using Pearson’s chi-square test or Fisher’s exact test, depending on whether cells with an expected frequency < 5 exceeded 20%. For the event of interest, the incidence was calculated based on the number of cases relative to the follow-up period in person-years, and the % Poisson CI was calculated [37]. A simple Cox proportional hazards regression model was fitted for individual factors, and multiple Cox proportional hazards regression models, including predetermined correction variables, were fitted for each analysis.

All statistical analyses were performed using SAS (version 9.4; SAS Institute Inc., Cary, NC), R (version 4.0.3; The R Foundation for Statistical Computing, Vienna, Austria). As a general rule, all tests were performed as two-sided tests with a significance level of 5%.

### Evaluation measure

To identify the effect size for the difference in statistics calculated from DB_III_ and DB_DII_, Cohen’s h was applied for percentage of categorical variables or distribution of attribute variables, and Cohen’s f was applied for the explanatory power of the regression model [38, 39]. However, these can be applied when comparing the difference between two independent groups, and because the data used in this study were considered to be data generated by independently linking two different datasets, it was assumed that independence was assured.

The effect size was interpreted as none if < 0.01, very small if ≥ 0.01 but < 0.2, small if ≥ 0.2 but < 0.5, medium if ≥ 0.5 but < 0.8, and large if ≥ 0.8. For a comprehensive comparison of the two datasets, the results from DB_III_ were classified as “good,” “poor,” and “insufficient” relative to the results from DB_DII_, as described below. First, if the effect size of the difference was < 0.5 or the estimates were similar, then such cases were defined as being “good” (○) based on the determination that there was no difference in the analysis results between the linkage levels. Second, if the effect size of the difference was ≥ 0.5 but < 0.8, the direction of the effect size was the same, but the values were over/underestimated or the direction of the effect size differed but was not significant. Accordingly, such cases were defined as “poor” (△) based on the determination that the results from different linkage levels are not similar. Third, if the effect size of the difference was ≥ 0.8, or the direction of the effect size was significantly estimated in opposite directions, then such cases were defined as “insufficient” (X) based on the determination that the results from the DB_III_-linked dataset are unreliable compared with DB_DII_.

## Results

### Linkage result

**Fig. 1:**
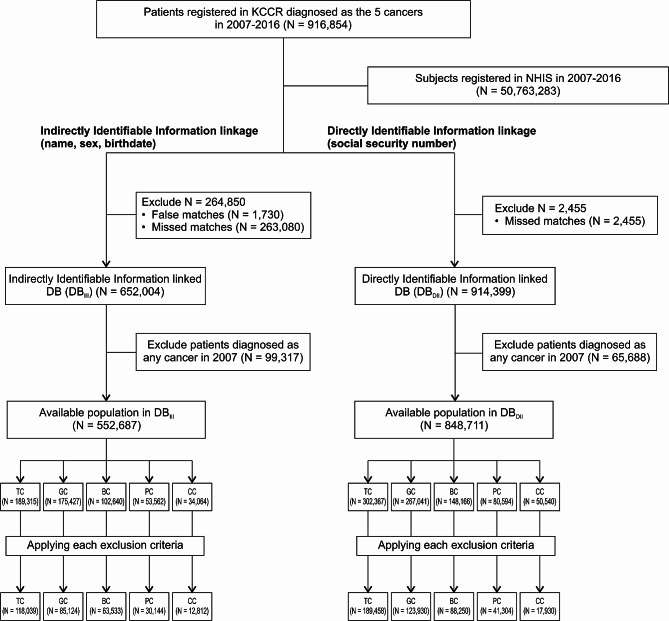
Flow chart for the linkage process

Figure 1 shows the results of linking NHIS data for 916,854 patients diagnosed with TC, GC, BC, PC, or CC at least once among all patients with cancer registered in the KCCR between 2007 and 2016. When the KCCR and national health insurance claims data were linked based on III (sex, name, and DOB), there were 1,730 (0.2%) false matches and 263,080 (28.7%) missed matches. When linked based on DB_DII_ (RRN), there were 2,455 (0.3%) missed matches. When these were excluded, DB_III_ had 652,004 patients, and DB_DII_ had 914,399 patients; thus, the linkage rate of DB_III_ relative to DB_III_ was 71.3%. To define new cancer cases, patients diagnosed with cancer disease codes starting with the letter “C” as of 2007 were excluded. The findings showed that the linkage of DB_III_ O relative to DB_DII_ was 65.1%, whereas the linkage rate by cancer type varied between a minimum of 62.6% and a maximum of 69.3%. When the analysis sets were extracted by applying the inclusion and exclusion criteria by cancer type, the TC results showed 118,039 patients with DB_III_ and 189,458 patients with DB_DII_. TC had the largest sample size among all the cancer types, followed by GC, BC, PC, and CC. The linkage rate according to the cancer type varied between 62.3% and 73.0%. subtotal gastrectomy.

### Descriptive statistics

The distribution of treatment groups by cancer type (use of vitamin D for TC, total gastrectomy or subtotal gastrectomy or ESD/EMR for GC, anti-hormone therapy for BC, ADT for PC, and (RT) for CC) was similar in DB_III_ and DB_DII_. Investigation of the distribution of treatment groups by strata according to sex and age group also showed a similar distribution in DB_III_ and DB_DII_. Further details are provided in the additional applicable information file (Supplementary Table 4).

To investigate the distribution of variables other than treatment groups by cancer type within the analysis set, the distribution of baseline characteristics in the two linked datasets was compared. In TC, the percentage of patients with CCI 0 or 1 was 17.2% in DB_III_ and 9.9% in DB_DII_ (Cohen’s h = 0.216). For BC, the percentage of patients who underwent the BMD test was 39.5% for DB_III_ and 56.6% for DB_DII_ (Cohen’s h = 0.343), showing a “small” difference. For all other variables, the effect size of the difference between DB_III_ and DB_DII_ was “none,” “almost none,” or “very small”. The additional applicable information shows this in more detail [see Supplementary Table 5].

### Group comparison

In the comparison of differences in the effect size between DB_III_ and DB_DII_ to determine whether there were differences in the results from comparing the baseline characteristics between the treatment groups by cancer type, the direction or the size of the difference between groups appeared slightly different for BMD test in BC and CCI ≤ 1 in TC that showed “small” effect size for the difference in descriptive statistics analysis (Cohen’s h = 0.222 for less than one CCI for TC; 0.337 for BMD test in BC). However, a comparison of the distribution of all other variables in the two linked datasets showed that the differences were “almost none” or “very small”. Further details are provided in the additional applicable information file (Supplementary Tables 6–10).

### Incidence rate estimation

A comparison of the total incidence of osteoporotic fractures between treatment groups by cancer type showed that the incidence of all fractures and fractures in different parts of the body was underestimated in DB_III_, as compared with DB_DII_ (Table 1). With regard to the incidence of all fractures, the incidence rates were estimated to be higher in the no vitamin D group in TC, ADT group in PC, and RT group in CC for both DB_III_ and DB_DII_. In GC, the frequency of fractures according to the type of surgery appeared in the order of total gastrectomy, subtotal gastrectomy, and ESD/EMR. In BC, the AI-only group showed the highest incidence of fractures, whereas the tamoxifen-only group showed the lowest incidence. Regarding the incidence of fractures in different parts of the body, the frequency of fractures appeared in the order of distal radius, vertebral, hip, and proximal humerus fractures in the TC, GC, and BC groups. In PC, however, a vertebral fracture was the most common, and proximal humerus fracture was the least common, with DB_III_ and DB_DII_ showing different orders for the frequency of hip and distal radius fractures. Further details are provided in the additional applicable information file (see Supplementary Table 11). Among these, CC that had a relatively smaller sample size, showed that incidence in the non-RT group was underestimated in DB_III_ compared with DB_DII_, whereas the incidence in the RT group was overestimated in DB_III_ compared with DB_DII_. Accordingly, the risk of RT being overestimated in DB_III_ was determined.

**Table 1:** Comparison of incidence rate estimation and treatment effect evaluation per linkage level

Treatment by Cancer Type	Incidence rate per 100,000 person-years (95% CI)				Adjusted HR* (95% CI)		
	DB_III_	DB_DII_	Effect size		DB_III_	DB_DII_	Effect size
Thyroid cancer							
No Vitamin D	1.3 (1.2–1.4)	1.5 (1.5–1.6)	0.02		1 (Reference)	1 (Reference)	
Vitamin D	1.1 (0.9–1.2)	1.4 (1.3–1.5)	0.00		0.83 (0.70–0.99)	0.83 (0.75–0.91)	0.00
Gastric cancer							
Total gastrectomy	2.6 (2.4–2.9)	3.2 (3.0–3.4)	0.02				
Subtotal gastrectomy	2.1 (2.0–2.2)	2.7 (2.6–2.8)	0.04		1.24 (1.10–1.39)	1.26 (1.15–1.37)	0.01
ESD/EMR	1.8 (1.6–1.9)	2.3 (2.2–2.5)	0.02		1 (Reference)	1 (Reference)	
Breast cancer							
Non-HT	2.2 (2.1–2.4)	2.2 (2.1–2.4)	0.00		1 (Reference)	1 (Reference)	
AI-only	3.5 (3.2–3.7)	3.6 (3.4–3.8)	0.00		0.95 (0.85–1.07)	1.03 (0.94–1.14)	0.04
TAM-only	1.2 (1.1–1.3)	1.2 (1.1–1.3)	0.00		0.77 (0.69–0.87)	0.80 (0.73–0.89)	0.02
AI + TAM	1.3 (0.6–2.4)	2.9 (1.9–3.9)	0.01		0.42 (0.21–0.85)	0.94 (0.66–1.35)	0.44
Prostate cancer							
Non-ADT	1.3 (1.2–1.4)	1.9 (1.8–2.1)	0.05		1 (Reference)	1 (Reference)	
ADT	4.1 (3.8–4.5)	5.4 (5.1–5.7)	0.04		2.14 (1.85–2.48)	1.96 (1.76–2.17)	0.05
Cervical cancer							
Non-RT	0.4 (0.3–0.5)	0.5 (0.4–0.6)	0.01		1 (Reference)	1 (Reference)	
RT	1.1 (0.8–1.6)	1.0 (0.7–1.3)	0.01		2.62 (1.63–4.23)	1.80 (1.18–2.73)	0.21

Abbreviations: ADT: androgen deprivation therapy; AI: aromatase inhibitors; CI: confidence interval; DB: data base; DII: directly identifiable information; III: indirectly identifiable information; ESD: Endoscopic submucosal dissection; EMR: Endoscopic mucosal resection; HR: hazard ratio; HT: hormone therapy; III: indirectly identifiable information; RT: radiation therapy

*Adjusted HR was computed after all covariates excluding the treatment group variable

### Cox PH regression model for treatment effect

To calculate the treatment effect on the incidence of osteoporotic fractures by cancer type, multiple Cox proportional hazards regression model was fitted to adjust the covariates, such as age, sex, and CCI. The treatment risks are shown in Table 1 and Supplementary Table 12. Relative to the DB_III_ results, the risk of fracture was lower by 0.83 (95% CI, 0.70–0.99) when using vitamin D after surgery as compared with not using vitamin D in TC cases. In BC cases, the risk of fracture was lower by 0.95 (95% CI, 0.85–1.07), 0.77 (95% CI, 0.69–0.87), and 0.42 (95% CI, 0.21–0.85) in the AI, Tamoxifen, and AI + Tamoxifen groups, respectively, as compared with the non-HT group. In GC cases, the risk of fracture was higher by 1.24 (95% CI, 0.10–1.39) in the gastrectomy group than in the ESD/EMR group. In PC cases, the risk of fracture was higher by 2.14 (95% CI, 1.85–2.48) in the ADT group than in the non-ADT group. In CC cases, the risk of fracture was higher by 2.62 (95% CI, 1.63–4.23) in the RT group than in the non-RT group. Risks in the same direction were derived using DB_DII_. Relative to DB_DII_, the risks in DB_III_ showed a “very small” effect size of all differences of < 0.2; however, the results confirmed that the treatment effect was overestimated in DB_III_ for treatment group of all cancer types, except TC.

### Subgroup analysis for the moderation effect

To investigate whether treatment effects would change according to other factors, such as age or cancer stage, differences between treatment groups according to age and summary staging in BC and PC cases were compared, the outcomes of which are shown in Table 2. In BC cases, using AIs increased the risk of fracture compared with non-HT in patients aged < 50 years, but decreased the risk in patients aged ≥ 50 years. Despite the contradictory effects, the results were not significant, and similar results were observed for both DB_III_ and DB_DII_. In PC cases, investigation of treatment effects according to summary staging revealed that in the localized group, the risk for fracture in the ADT group in DB_III_ and DB_DII_ was 2.27 (95% CI, 1.91–2.70) and 1.92 (95% CI, 1.70–2.17), respectively, showing that the risk was overestimated in DB_III_. In the regional group, the risk was 1.80 (95% CI, 1.37–2.36) in DB_III_ and 2.05 (1.67–2.52) in DB_DII_, confirming that it was underestimated.

**Table 2:** Comparison of the result of moderation effect evaluation by subgroup analysis

Subgroup by cancer type	Adjusted HR* (95% CI)				Adjusted HR* (95% CI)		
	DB_III_	DB_DII_	Effect size		DB_III_	DB_DII_	Effect size
Breast cancer	In age < 50				In age ≥ 50		
Non-HT	1 (Reference)	1 (Reference)			1 (Reference)	1 (Reference)	
AI-only	1.31 (0.94–1.84)	1.21 (0.85–1.71)	0.04		0.89 (0.79–1.01)	0.98 (0.88–1.08)	0.05
TAM-only	0.83 (0.69–1.01)	0.93 (0.78–1.10)	0.06		0.80 (0.68–0.94)	0.78 (0.69–0.89)	0.01
AI + TAM	0.19 (0.01–3.03)	0.27 (0.04–1.95)	0.19		0.46 (0.23–0.92)	0.98 (0.68–1.42)	0.42
Prostate cancer	Localized stage				Regional stage		
Non-ADT	1 (Reference)	1 (Reference)			1 (Reference)	1 (Reference)	
ADT	2.27 (1.91–2.70)	1.92 (1.70–2.17)	0.09		1.80 (1.37–2.36)	2.05 (1.67–2.52)	0.07

Abbreviations: ADT: androgen deprivation therapy; AI: aromatase inhibitors; CI: confidence interval; DB: data base; DII: directly identifiable information; III: indirectly identifiable information; HR: hazard ratio; HT: hormone therapy; III: indirectly identifiable information

*Adjusted HR was computed after all covariates excluding the treatment and subgroup variables

### Synthetic result

When the results from analyses using unique DB_DII_-linked data and DB_III_-linked data were classified into three levels (good, poor, and insufficient), the results for BC cases showed that the treatment effect in treatment effect evaluation appeared in the opposite direction. However, because the results were not significant, it was classified as poor research level. In CC cases, the treatment effect size in incidence and treatment effect evaluations was overestimated. Thus, it was determined to be a poor research level. In PC cases, analysis of the moderation effect for identification of the risk of fracture with ADT according to summary staging showed conflicting over/underestimation of the risk. Hence, the research level was assessed to be “insufficient” (Table 3).

**Table 3:** Synthetic result and related research scope of III linkage compared with DII linkage

Analysis method	Study level of each topic*					Related research scope
	TC	GC	BC	PC	CC	
Descriptive statistics	○	○	○	○	○	Epidemiologic indices including prevalence or cross-sectional proportion
Group comparison	○	○	○	○	○	Comparison of characteristics by the factor of interest
Incidence rate estimation	○	○	○	○	△	Incidence and cumulative survival rates over time
Cox PH regression model	○	○	△	○	△	Evaluation of treatment effect, risk factor exploration, development of prediction model, etc.
Subgroup analysis	-	-	△	X	-	Moderation effect evaluation by subgroup analysis or the interaction effect testing

Abbreviations: III: indirectly identifiable information; DII: directly identifiable information; TC: thyroid cancer; GC: gastric cancer; BC: breast cancer; PC: prostate cancer; CC: cervical cancer; PH: proportional hazard

*Study level was divided into three categories: O = good, △ = insufficient, and X = poor

## Discussion

We identified the characteristics and limitations of the analysis results according to the data linkage level in big-data-based health and medical research. Accordingly, linkage levels appropriate for analysis topics and sample sizes were tested on the basis of the empirical analysis of the incidence of osteoporotic fracture, which is a metabolic disorder that accompanies patients with cancer.

The accuracy of the descriptive statistics and group comparison analyses was dependent on the sample size. In the comparison of the distribution of baseline characteristics according to epidemiological indicators, such as prevalence and cross-sectional incidence, or variables of interest, disease groups with sufficient sample sizes, such as TC, GC, and BC, showed good results even if the level of identified information needed for data linkage was not that high. In CC cases that had the smallest sample size, descriptive statistics and group comparison analysis results were at a good level, but the sample size was less than 1,000 in certain categories, such as age group and insurance type. Therefore, when the sample size for each category of FOI is too small, the linkage level should be increased to ensure sufficient statistical power, which can prevent distorted results.

In the incidence analysis, the results showed a “poor” research level for CC that was determined to be the effect of an absolutely small sample size. However, for incidence considering time, it is difficult to assess the sample size alone. Therefore, the linkage level could vary depending on whether a sufficient follow-up period can be assured and the expected incidence of the event of interest.

In the regression models for the treatment effect evaluation, a poverty-poor research level was found for BC and CC. In CC cases, distribution by group or distribution of baseline characteristics appeared differently owing to the small sample size. Therefore, regression models based on such variables would produce results that are over or underestimated. Although not significant, BC cases that showed an effect size in opposite directions showed relatively low levels of 22.34% and 0.77% in the AI and combination therapy groups, respectively. Therefore, even if the total sample size is sufficiently large, it is necessary to have a sufficient sample size for each level of the variable of interest in BC. Thus, when evaluating the treatment effect, identifying the risk factors, or developing the prediction models, the total sample size for the variables included in the models and the sample size for each category of the variables of interest should be reviewed. If a sufficient sample size is secured, a “good” level of research results can be expected, even with indirectly information-linked data.

In the analysis of the moderated mediation effect, to assess whether the effect of the FOI varied according to the parameters or subgroups, an “insufficient” research level was derived in PC cases. Despite having at least 30% of patients per summary staging category, an increase or decrease in the treatment effect according to summary staging appeared contrary. This notion cannot be attributed simply to a problem with sample size; instead, it is necessary to check the characteristics of patients who were omitted when linking III.

DB_III_-linked data and unique DB_DII_-linked data were analyzed on different servers owing to space constraints. Consequently, it was impossible to identify patients who were omitted during the data linkage process; therefore, this study has the limitation of not being able to conduct additional analysis of the characteristics of omitted patients. In the future, it will be necessary to identify the cause of the differences in moderation effects by evaluating the baseline characteristics of patients who were omitted during the data linkage process.

This study also confirmed the limitation of not being able to guarantee 100% accuracy, even when a unique DII was used for inter-agency data linkage. When planning a study with a small size, because it is a rare disease or a topic with a narrow scope, it is necessary to assume that analysis could become impossible owing to the omission of information. Accordingly, it is necessary for the agencies providing the data to sufficiently consult the researchers in advance about the percentage for each category and the percentage of an event of interest by category that meets the study objectives, such as the entire study population, age groups, and comorbidities. Moreover, a preliminary review report based on sample size and statistical power should be drafted and reviewed to design the best possible environment for providing a unique DII linked to datasets. Additionally, when researchers present articles or reports on epidemiological indicators, such as incidence rate, based on link data, they are recommended to provide information, such as linkage rate and group percentage before and after linkage. We incorporated the expertise of professionals from each institution that provided data for our study through two expert forums. Owing to the absence of a large-scale expert-opinion gathering process, there were limitations in constructing checklists similar to RECORD or STROSA [40–42]. We plan to address this limitation in future research.

While the primary focus of this study was to compare the effect sizes of two linkage methods, it is important to acknowledge the limitation regarding the absence of propensity score matching results for comparison. Propensity score matching was not conducted for the linkage data as it diverged from the main objective. However, recognizing the significance of this methodological approach, we plan to address this limitation in future research. By conducting a more comprehensive comparison between the original dataset and the propensity-score-matched dataset, which is categorized by each type of cancer and specific topics, we aim to provide further insights into the effectiveness of different linkage methods and their implications for cancer research.

## Conclusions

Two different datasets based on DB_III_ and unique DB_DII_ were linked to conduct an empirical analysis of the results. Considering linkage errors that occur during the data linkage process, it is important to ensure that the patient population for each disease and sample size for each FOI are large enough, while the linkage level could vary depending on the size of statistical power. In the future, we should make a study plan with diverse linkage methods depending on the research purpose, with an exact sample size for each disease type. Through such efforts, it is expected that agencies and researchers can be used as references when setting the data linkage level.

### Electronic supplementary material

Below is the link to the electronic supplementary material.

Supplementary Material 1

## Data Availability

The datasets generated and/or analyzed during the current study are not publicly available because NHIS Korea allows only authorized persons to access data. However, data are available from the corresponding author (JKB) upon reasonable request.

## Abbreviations

DII: Directly identifiable information
III: Indirectly identifiable information
DOB: Date of birth
RRN: Resident registration number
DB: Data base
CC: Cervical cancer
PC: Prostate cancer
GC: Gastric cancer
TC: Thyroid cancer
BC: Breast cancer
SEER: Surveillance, epidemiology, and end results
HR: Hazard ratio
CI: Confidence interval
FOI: Factor of interest
RT: Radiation therapy
ADT: Androgen deprivation therapy
MoHW: Ministry of Health and Welfare
NHIS: National Health Insurance Service
HIRA: Health Insurance Review and Assessment Service
KDCA: Korea Disease Control and Prevention Agency
NCC: National Cancer Center
KCCR: Korea Central Cancer Registry
ESD: Endoscopic submucosal dissection
EMR: Endoscopic mucosal resection
HT: Hormone therapy
AIs: Aromatase inhibitors
CCI: Charlson Comorbidity Index
BMD: Bone mineral density
