# Supplementary material for: Impact of linkage level on inferences from big data analyses in health and medical research: an empirical study
Source: BMC Med Inform Decis Mak. 2024 Jul 9;24:193. doi: 10.1186/s12911-024-02586-0 (PMC11234607; doi:10.1186/s12911-024-02586-0)
Supplement: Supplementary file 1 — Supplementary Material 1 [file 12911_2024_2586_MOESM1_ESM.docx]

# Supplementary Appendix

## Supplementary Table 1. Inclusion/Exclusion criteria and definition of treatment by cancer type

| Type | Inclusion Criteria | Exclusion Criteria | Definition of Treatment |
| --- | --- | --- | --- |
| Thyroid cancer | Adults over the age of 18 who were first diagnosed with thyroid cancer and underwent total thyroidectomy between 2008 and 2016 | - No diagnosis of cancer on the NHIS database  - Second cancer diagnosed after the index date  - SEER summary stage of “Distant” or “Unstageable”  - Death within six months after the index date  - Prescription of osteoporosis medications before the index date  - Osteoporotic fracture before the index date  - Chronic kidney disease diagnosed as the main disease with triple or more outpatient visits or two days or more of hospitalization during the whole observation period  - Hypoparathyroidism diagnosed as the main disease with triple or more outpatient visits or two days or more of hospitalization before the index date  - Parathyroidectomy surgery before the index date  - Prescription of Levothyroxine or active vitamin D before the index date | Hypoparathyroidism treatment was defined as three or more times of prescriptions of active vitamin D for 90 days or more within one year after the index date |
| Gastric cancer | Adults over the age of 18 years who were first diagnosed with gastric cancer and underwent total gastrectomy, subtotal gastrectomy, or ESD/EMR surgery between 2008 and 2016 | - No diagnosis of cancer on the NHIS database  - Second cancer diagnosed after the index date  - SEER summary stage of “Distant” or “Unstageable”  - Death within six months after the index date  - Prescription of osteoporosis medications before the index date  - Osteoporotic fracture before the index date  - Rheumatoid arthritis diagnosed before the index date  - Prescription of glucocorticoid for more than 90 days before the index date | The first surgery after the index date |
| Breast cancer | Adults over the age of 18 years who were first diagnosed with invasive breast cancer and underwent mastectomy or breast-conserving surgery between 2008 and 2016 | - No diagnosis of cancer on the NHIS database  - Second cancer diagnosed after the index date  - SEER summary stage of “Distant” or “Unstageable”  - Death within 6 months after the index date  - Prescription of osteoporosis medications before the index date  - Osteoporotic fracture before the index date  - Rheumatoid arthritis diagnosed before the index date  - Prescription of glucocorticoid for more than 90 days before the index date  - Diagnosis of breast carcinoma in situ before the index date | Hormone therapy was defined as the prescription of Aromatase inhibitor (AI) or Tamoxifen (TAM) for 90 days or more within 1 year after the index date |
| Prostate cancer | Adults over the age of 18 years who were first diagnosed with prostate cancer between 2008 and 2016 | - No diagnosis of cancer on the NHIS database  - Second cancer diagnosed after the index date  - SEER summary stage of “Distant” or “Unstageable”  - Death within 6 months after the index date  - Prescription of osteoporosis medications before the index date  - Osteoporotic fracture before the index date  - Rheumatoid arthritis diagnosed before the index date  - Prescription of glucocorticoid for more than 90 days before the index date  - Prescription of antiandrogen for 1 to 89 days after the index date | Androgen deprivation therapy (ADT) was defined as the 90 days or more of prescription of antiandrogen medication or more than once GnRH drug within 1 year after the index date |
| Cervical cancer | Adults over the age of 18 years who were first diagnosed with cervical cancer between 2008 and 2016 | - No diagnosis of cancer on the NHIS database  - Second cancer diagnosed after the index date  - SEER summary stage of “Distant” or “Unstageable”  - Death within 6 months after the index date  - Prescription of osteoporosis medications before the index date  - Osteoporotic fracture before the index date  - Rheumatoid arthritis diagnosed before the index date  - Pelvic fracture before the index date | Radiation therapy (RT) was defined as 20 times or more of the radiation treatment code within 1 year after the index date |

## Supplementary Table 2. Operational definition of outcome and covariates.

| Variable | Definition |
| --- | --- |
| Outcome |  |
| Hip fracture | (1) Diagnosis + Procedure + Hospitalization or (2) Procedure + Emergency room visit |
| Vertebral fracture | (1) Diagnosis + Procedure, (2) Hospitalization with diagnosis as main disease, or (3) Vertebral imaging code with diagnosis as the main disease |
| Distal radius fracture | (1) Diagnosis + Procedure, or (2) Distal radius conserving procedure with diagnosis as the main disease |
| Proximal humerus fracture | (1) Diagnosis + Procedure, or (2) Proximal humerus conserving procedure with diagnosis as the main disease |
| Pelvic fracture | Diagnosis + Hospitalization |
| Covariates |  |
| Osteoporotic medication | More than one prescription of medication within one year after the index date |
| Bone mass density test | More than one procedure of medication within one year after the index date |
| Radiation therapy | More than one procedure within one year after the index date |

## Supplementary Table 3. Code of diagnosis, procedure, and drug in the NHIS database for the operational definition of variables.

| Cancer Type | Term | Category | Code | Weight* |
| --- | --- | --- | --- | --- |
| Thyroid cancer | Thyroid cancer | Diagnosis | C73x |  |
|  | Thyroidectomy | Procedure | P4552, P4554, P4561 |  |
|  | Hypoparathyroidism | Diagnosis | E209 |  |
|  | Parathyroidectomy | Procedure | P4541, P4542, P4543 |  |
|  | Active Vitamin D | Drug | 121601ACS, 104601ACS, 104601ATB, 104602ACS, 468000ATE, 121401ACS, 121402ACS, 121401ATB, 121402ATB |  |
|  | Levothyroxine | Drug | 183601ATB, 296800ATB |  |
| Gastric cancer | Gastric cancer | Diagnosis | C16x |  |
|  | Total gastrectomy | Procedure | QA536, Q2533, Q2536, Q2534, Q2537 |  |
|  | Subtotal gastrectomy | Procedure | Q2571, Q2572, Q2573, Q0259, Q2594, Q0251, Q0252, Q0203, Q0254, Q0255, Q0256, Q0257, Q0258, Q2598, Q2601 |  |
|  | ESD/EMR | Procedure | QZ933, QX704, Q7652, QX701 |  |
| Breast cancer | Breast cancer | Diagnosis | C50x |  |
|  | Breast carcinoma in situ | Diagnosis | D050, D051, D059 |  |
|  | Mastectomy | Procedure | N7131, N7132, N7133, N7134, N7135, N7136, N7137, N7138, N7139, P2121, P2122, P2123, P2124, N7121, N7122 |  |
|  | Breast-conserving surgery | Procedure | N7140, N7141, N7142, N7143, N7144, N7145, N7146, N7147, N7148, N7149, N7150, N7151, N7152, N7153 |  |
|  | Aromatase inhibitor (AI) | Drug | 109001ATB, 182201ATB, 358401ATB |  |
|  | Tamoxifen (TAM) | Drug | 234501ATB, 234502ATB |  |
| Prostate cancer | Prostate cancer | Diagnosis | C61x |  |
|  | Antiandrogen therapy | Drug | 139401ATB, 155402ATB, 159001ATB, 117201ATB, 117202ATB, 162101ATB |  |
|  | GnRH | Drug | 167201BIJ, 167202BIJ, 182601BIJ, 182602BIJ, 182603BIJ, 182604BIJ, 182605BIJ, 182606BIJ, 182607BIJ, 182608BIJ, 182609BIJ, 182610BIJ, 244901BIJ, 244902BIJ, 420701BIJ, 467501BIJ |  |
| Cervical cancer | Cervical cancer | Diagnosis | C530, C539 |  |
|  | Pelvic fracture | Diagnosis | M484, M483, M489, S321, S323, S324, S325, S327, S328, S329 |  |
|  | Radiation therapy (RT) | Procedure | HD014, HD018, HD031, HD032, HD033, HD041, HD061, HD418, HD441, HY402, HZ271 |  |
| All | Chronic kidney disease | Diagnosis | N185 |  |
|  | Rheumatoid arthritis | Diagnosis | M05x, M06x |  |
|  | Glucocorticoid | Drug | 116401ATB, 116501ATB, 296900ATB, 141901ATB, 141903ATB, 141904ATB, 193301ATB, 193302ATB, 193303ATB, 193304ATB, 193305ATB, 217401ATB, 217001ATB, 622901ATR, 622902ATR, 622903ATR, 243201ATB, 243202ATB, 243203ATB, 243311ATC, 170901ATB, 170905ATB, 170906ATB, 140801ATB, 140802ATB |  |
|  | Osteoporotic medication | Drug | 151101BIJ, 151102BIJ, 151103BIJ, 151130BIJ, 151131BIJ, 640100ATB, 674500ATB, 617101ATB, 659200ACH, 659200ATB, 698200ATB, 358001ATB, 358002ATB, 487502BIJ, 646301BIJ |  |
|  | Hip fracture | Diagnosis | S720, S721 |  |
|  | Hip fracture | Procedure | N0601, N0991, N0981, N0641, N0652, N0654, N0715, N0711, N0611, N2070, N2710 |  |
|  | Vertebral fracture | Diagnosis | M484, M485, M495, M808, S220, S221, S320, S327, T080 |  |
|  | Vertebral fracture | Procedure | N0471, N0472, N0473, N0474, N0630 |  |
|  | Vertebral imaging | Procedure | G430, G440, G450, G460 |  |
|  | Distal radius fracture | Diagnosis | S525, S526 |  |
|  | Distal radius fracture | Procedure | N1601, N1611, N1603, N1613, N0996, N0998, N0983, T6020, T6030, T6151, T6152 |  |
|  | Proximal humerus fracture | Diagnosis | S422, S423 |  |
|  | Proximal humerus fracture | Procedure | N0602, N0612, N0992, N0982, N0986, N0722, N2711, N2716, T6010, T6110 |  |
|  | Pelvic fracture | Diagnosis | M484, M483, M489, S321, S323, S324, S325, S327, S328, S329 |  |
|  | Bone mass density test | Procedure | HC341, HC342, HC343, HC344, HC345, HC346 |  |
| CCI | Myocardial infarction | Diagnosis | I21x, I22x, I252 | 1 |
|  | Congestive heart failure | Diagnosis | I099, I110, I130, I132, I255, I420, I425-I429, I43x, I50x, I290 | 1 |
|  | Peripheral vascular disease | Diagnosis | I70x, I71x, I731, I738, I739, I771, I790, I792, K551, K558, K559, Z958, Z959 | 1 |
|  | Cerebral vascular accident | Diagnosis | G45x, G48x, H340, I60x-I69x | 1 |
|  | Dementia | Diagnosis | F00x-F03x, F051, G30x, G311 | 1 |
|  | Chronic pulmonary disease | Diagnosis | I278, I279, J40x-J47x, J60x-J67x, J684, J701, J703 | 1 |
|  | Rheumatologic disease | Diagnosis | M05x, M06x, M315, M32x-M34x, M351, M353, M360 | 1 |
|  | Peptic ulcer disease | Diagnosis | K25x-K28x | 1 |
|  | Mild liver disease | Diagnosis | B18x, K700-K703, K709, K713-K715, K717, K73x, K74x, K760, K762-K764, K768, K769, Z944 | 1 |
|  | Diabetes without chronic complication | Diagnosis | E100, E101, E106, E108, E109, E110, E111, E116, E118, E119, E120, E121, E126, E128, E129, E130, E131, E136, E138, E139, E140, E141, E146, E148, E149 | 1 |
|  | Diabetes with chronic complication | Diagnosis | E102-E105, E107, E112-E115, E117, E122-E125, E127, E132-E135, E137, E142-E145, E147 | 2 |
|  | Herriplegia or paraplegia | Diagnosis | G041, G114, G801, G802, G81x, G82x, G830-G834, G839 | 2 |
|  | Renal disease | Diagnosis | I120, I131, N032-N037, N052-N057, N18x, N19x, N250, Z490-Z492, Z992 | 2 |
|  | Any malignancy including leukemia and lymphoma | Diagnosis | C00x-C26x, C30x-C34x, C37x-C41x, C43x, C45x-C58x, C60x-C76x, C81x-C85x, C88x, C90x-C97x | 2 |
|  | Moderate or severe liver disease | Diagnosis | I850, I859, I864, I982, K704, K711, K721, K729, K765, K766, K767 | 3 |
|  | Metastatic solid tumor | Diagnosis | C77x - C80x | 6 |
|  | Acquired immune deficiency syndrome/HIV | Diagnosis | B20x-B22x, B24x | 6 |

Abbreviation: CCI, Charlson Comorbidity Index; HIV, human immunodeficiency virus.

The code for diagnosis follows the ICD-10 code (International Classification of Diseases, 10th version).

*Weight was applied to compute the CCI based on the modified CCI by Quan et al. (2005).

## Supplementary Table 4. Distribution of treatment group by cancer type per linkage level.

|  | Thyroid Cancer | |  | Gastric Cancer | |  | Breast Cancer | |  | Prostate Cancer | |  | Cervical Cancer | |  |
| --- | --- | --- | --- | --- | --- | --- | --- | --- | --- | --- | --- | --- | --- | --- | --- |
|  | DB_III_ | DB_DII_ | Effect Size | DB_III_ | DB_DII_ | Effect size | DB_III_ | DB_DII_ | Effect Size | DB_III_ | DB_DII_ | Effect Size | DB_III_ | DB_DII_ | Effect Size |
| Group* | (N=118,039) | (N=189,458) |  | (N=85,124) | (N=123,930) |  | (N=63,533) | (N=88,250) |  | (N=28,490) | (N=39,154) |  | (N=12,812) | (N=17,930) |  |
| Total |  |  |  |  |  |  |  |  |  |  |  |  |  |  |  |
| Group1 | 109247 (92.6%) | 167305 (88.3%) | 0.14 | 14428 (17.0%) | 20940 (16.9%) | 0.00 | 20188 (31.8%) | 25325 (28.7%) | 0.07 | 16794 (59.0%) | 20881 (53.3%) | 0.11 | 10059 (78.5%) | 14234 (79.4%) | 0.02 |
| Group2 | 8792 (7.5%) | 22153 (11.7%) |  | 52572 (61.8%) | 77002 (62.1%) | 0.01 | 13764 (21.7%) | 19711 (22.3%) | 0.01 | 11696 (41.1%) | 18273 (46.7%) |  | 2753 (21.5%) | 3696 (20.6%) |  |
| Group3 | - | - |  | 18124 (21.3%) | 25988 (21.0%) | 0.01 | 29220 (46.0%) | 42538 (48.2%) | 0.04 | - | - |  | - | - |  |
| Group4 | - | - |  | - | - |  | 361 (0.6%) | 676 (0.8%) | 0.02 | - | - |  | - | - |  |
| Male |  |  |  |  |  |  |  |  |  |  |  |  |  |  |  |
| Group1 | 17940 (93.8%) | 32147 (90.2%) | 0.13 | 10736 (16.8%) | 15608 (16.9%) | 0.00 | - | - |  | 16794 (59.0%) | 20881 (53.3%) | 0.11 | - | - |  |
| Group2 | 1179 (6.2%) | 3475 (9.8%) |  | 38196 (59.6%) | 55606 (60.0%) | 0.01 | - | - |  | 11696 (41.1%) | 18273 (46.7%) |  | - | - |  |
| Group3 | - | - |  | 15173 (23.7%) | 21426 (23.1%) | 0.01 | - | - |  | - | - |  | - | - |  |
| Group4 | - | - |  | - | - |  | - | - |  | - | - |  | - | - |  |
| Female |  |  |  |  |  |  |  |  |  |  |  |  |  |  |  |
| Group1 | 91307 (92.3%) | 135158 (87.9%) | 0.15 | 3692 (17.6%) | 5332 (17.0%) | 0.02 | 20188 (31.8%) | 25325 (28.7%) | 0.07 | - | - |  | 10059 (78.5%) | 14234 (79.4%) | 0.02 |
| Group2 | 7613 (7.7%) | 18678 (12.1%) |  | 14376 (68.4%) | 21396 (68.4%) | 0.00 | 13764 (21.7%) | 19711 (22.3%) | 0.01 | - | - |  | 2753 (21.5%) | 3696 (20.6%) |  |
| Group3 | - | - |  | 2951 (14.0%) | 4562 (14.6%) | 0.02 | 29220 (46.0%) | 42538 (48.2%) | 0.04 | - | - |  | - | - |  |
| Group4 | - | - |  | - | - |  | 361 (0.6%) | 676 (0.8%) | 0.02 | - | - |  | - | - |  |
| Age ≤30s |  |  |  |  |  |  |  |  |  |  |  |  |  |  |  |
| Group1 | 22375 (93.9%) | 38219 (89.6%) | 0.16 | - | - |  | 3183 (36.8%) | 4370 (35.7%) | 0.02 | - | - |  | - | - |  |
| Group2 | 1467 (6.2%) | 4447 (10.4%) |  | - | - |  | 13 (0.2%) | 20 (0.2%) | 0.00 | - | - |  | - | - |  |
| Group3 | - | - |  | - | - |  | 5463 (63.1%) | 7848 (64.1%) | 0.02 | - | - |  | - | - |  |
| Group4 | - | - |  | - | - |  | 1  (0.0%) | 4 (0.0%) | 0.00 | - | - |  | - | - |  |
| Age 40s |  |  |  |  |  |  |  |  |  |  |  |  |  |  |  |
| Group1 | 35672 (92.8%) | 50344 (88.6%) | 0.15 | - | - |  | 7269 (27.0%) | 8425 (23.5%) | 0.08 | 236 (7.5%) | 75 (20.0%) | 0.37 | - | - |  |
| Group2 | 2779 (7.2%) | 6483 (11.4%) |  | - | - |  | 1118 (4.2%) | 1268 (3.5%) | 0.04 | 2933 (92.6%) | 301 (80.1%) |  | - | - |  |
| Group3 | - | - |  | - | - |  | 18431 (68.5%) | 26076 (72.6%) | 0.09 | - | - |  | - | - |  |
| Group4 | - | - |  | - | - |  | 80 (0.3%) | 143 (0.4%) | 0.02 | - | - |  | - | - |  |
| Age 50s |  |  |  |  |  |  |  |  |  |  |  |  |  |  |  |
| Group1 | 34834 (91.9%) | 49708 (87.8%) | 0.14 | - | - |  | 6898 (35.6%) | 8539 (32.1%) | 0.07 | 2933 (75.9%) | 3265 (72.7%) | 0.07 | - | - |  |
| Group2 | 3091 (8.2%) | 6921 (12.2%) |  | - | - |  | 7983 (41.2%) | 10782 (40.5%) | 0.01 | 930 (24.1%) | 1225 (27.3%) |  | - | - |  |
| Group3 | - | - |  | - | - |  | 4320 (22.3%) | 6935 (26.1%) | 0.09 | - | - |  | - | - |  |
| Group4 | - | - |  | - | - |  | 192 (1.0%) | 349 (1.3%) | 0.03 | - | - |  | - | - |  |
| Age ≥60s |  |  |  |  |  |  |  |  |  |  |  |  |  |  |  |
| Group1 | 16366 (91.8%) | 29034 (87.1%) | 0.15 | - | - |  | 2838 (33.1%) | 3991 (29.6%) | 0.08 | 13625 (56.0%) | 17315 (50.5%) | 0.11 | - | - |  |
| Group2 | 1455 (8.2%) | 4302 (12.9%) |  | - | - |  | 4650 (54.2%) | 7641 (56.6%) | 0.05 | 10711 (44.0%) | 16973 (49.5%) |  | - | - |  |
| Group3 | - | - |  | - | - |  | 1006 (11.7%) | 1679 (12.4%) | 0.02 | - | - |  | - | - |  |
| Group4 | - | - |  | - | - |  | 88 (2.1%) | 180 (1.3%) | 0.06 | - | - |  | - | - |  |

*Group definition by cancer type.

- group1=no vitamin D, group2=vitamin D for thyroid cancer.

- group1=total gastrectomy, group2=subtotal gastrectomy, group3=ESD/EMR for gastric cancer.

- group1=non-HT, group2=AI-only, group3=TAM-only, group4=both for breast cancer.

- group1=non-ADT, group2=ADT for prostate cancer.

- group1=non-radiation therapy (RT), group2=RT for cervical cancer.

**Supplementary Table 5.** Distribution of baseline characteristics per linkage level.

|  | Thyroid Cancer | |  | Gastric Cancer | |  | Breast Cancer | |  | Prostate Cancer | |  | Cervical Cancer | |  |
| --- | --- | --- | --- | --- | --- | --- | --- | --- | --- | --- | --- | --- | --- | --- | --- |
|  | DB_III_ | DB_DII_ | Effect Size | DB_III_ | DB_DII_ | Effect Size | DB_III_ | DB_DII_ | Effect Size | DB_III_ | DB_DII_ | Effect Size | DB_III_ | DB_DII_ | Effect Size |
| Variable | (N=118,039) | (N=189,458) |  | (N=85,124) | (N=123,930) |  | (N=63,533) | (N=88,250) |  | (N=28,490) | (N=39,154) |  | (N=12,812) | (N=17,930) |  |
| Sex |  |  |  |  |  |  |  |  |  |  |  |  |  |  |  |
| Male | 19119 (16.2%) | 35622 (18.8%) | 0.07 | 64105 (75.3%) | 92640 (74.8%) | 0.01 |  |  |  | 28490 (100.0%) | 39154 (100.0%) |  |  |  |  |
| Female | 98920 (83.8%) | 153836 (81.2%) |  | 21019 (24.7%) | 31290 (25.3%) |  | 63533 (100.0%) | 88250 (100.0%) |  |  |  |  | 12812 (100.0%) | 17930 (100.0%) |  |
| Age at diagnosis (year) |  |  |  |  |  |  |  |  |  |  |  |  |  |  |  |
| <40 | 23842 (20.2%) | 42666 (22.5%) | 0.06 | 3299 (3.9%) | 5387 (4.4%) | 0.02 | 8660 (13.6%) | 12242 (13.9%) | 0.01 | - | - |  | 2843 (22.2%) | 4037 (22.5%) | 0.01 |
| 40–49 | 38451 (32.6%) | 56827 (30.0%) | 0.06 | 12401 (14.6%) | 18671 (15.1%) | 0.01 | 26898 (42.3%) | 35912 (40.7%) | 0.03 | 291 (1.0%) | 376 (1.0%) | 0.01 | 4306 (33.6%) | 5727 (31.9%) | 0.04 |
| 50–59 | 37925 (32.1%) | 56629 (29.9%) | 0.05 | 26591 (31.2%) | 35598 (28.7%) | 0.06 | 19393 (30.5%) | 26605 (30.2%) | 0.01 | 3863 (13.6%) | 4490 (11.5%) | 0.06 | 3250 (25.4%) | 4393 (24.5%) | 0.02 |
| 60–69 | 17821 (15.1%) | 33336 (17.6%) | 0.07 | 26508 (31.1%) | 36682 (29.6%) | 0.03 | 6592 (10.4%) | 9797 (11.1%) | 0.02 | 11904 (41.8%) | 14698 (37.5%) | 0.09 | 1477 (11.5%) | 2201 (12.3%) | 0.02 |
| 70–79 |  |  |  | 14868 (17.5%) | 23882 (19.3%) | 0.05 | 1990 (3.1%) | 3694 (4.2%) | 0.06 | 10780 (37.8%) | 15970 (40.8%) | 0.06 | 936 (7.3%) | 1572 (8.8%) | 0.05 |
| ≥80 |  |  |  | 1457 (1.7%) | 3710 (3.0%) | 0.09 |  |  |  | 1652 (5.8%) | 3620 (9.3%) | 0.13 | - | - |  |
| Calendar year at diagnosis |  |  |  |  |  |  |  |  |  |  |  |  |  |  |  |
| 2008–2010 | 38028 (32.2%) | 61626 (32.5%) | 0.01 | 27060 (31.8%) | 40288 (32.5%) | 0.02 | 19486 (30.7%) | 27120 (30.7%) | 0.00 | 8240 (28.9%) | 12276 (31.4%) | 0.05 | 4863 (38.0%) | 6948 (38.8%) | 0.02 |
| 2011–2013 | 53859 (45.6%) | 86503 (45.7%) | 0.00 | 30663 (36.0%) | 44296 (35.7%) | 0.01 | 22013 (34.7%) | 30164 (34.2%) | 0.01 | 10110 (35.5%) | 13959 (35.7%) | 0.00 | 4250 (33.2%) | 5889 (32.8%) | 0.01 |
| 2014–2016 | 26152 (22.2%) | 41329 (21.8%) | 0.01 | 27401 (32.2%) | 39346 (31.8%) | 0.01 | 22034 (34.7%) | 30966 (35.1%) | 0.01 | 10140 (35.6%) | 12919 (33.0%) | 0.06 | 3699 (28.9%) | 5093 (28.4%) | 0.01 |
| Region |  |  |  |  |  |  |  |  |  |  |  |  |  |  |  |
| Urban | 88761 (75.2%) | 96275 (50.8%) | 0.08 | 58338 (68.5%) | 82392 (66.5%) | 0.04 | 48971 (76.7%) | 66453 (75.3%) | 0.03 | 20395 (71.6%) | 27350 (69.9%) | 0.04 | 9284 (72.5%) | 12644 (70.5%) | 0.04 |
| Rural | 29278 (24.8%) | 93183 (49.2%) |  | 26786 (31.5%) | 41538 (33.5%) |  | 14562 (23.3%) | 21797 (24.7%) |  | 8095 (28.4%) | 11804 (30.2%) |  | 3528 (27.5%) | 5286 (29.5%) |  |
| Insurance type |  |  |  |  |  |  |  |  |  |  |  |  |  |  |  |
| Local | 35253 (29.9%) | 70816 (37.4%) | 0.16 | 28248 (33.2%) | 40442 (32.6%) | 0.01 | 21620 (34.0%) | 30430 (34.48%) | 0.01 | 8613 (30.2%) | 11548 (29.5%) | 0.02 | 5194 (40.5%) | 7380 (41.2%) | 0.01 |
| Employment | 80649 (68.3%) | 115248 (60.8%) | 0.16 | 53686 (63.1%) | 79785 (64.4%) | 0.03 | 39966 (62.9%) | 55775 (63.2%) | 0.01 | 18966 (66.6%) | 26365 (67.3%) | 0.02 | 6975 (54.4%) | 9886 (55.1%) | 0.01 |
| Medical aids | 2137 (1.8%) | 3394 (1.8%) | 0.00 | 3190 (3.8%) | 3650 (3.0%) | 0.05 | 1947 (3.1%) | 2003 (2.27%) | 0.05 | 911 (3.2%) | 1151 (2.9%) | 0.02 | 643 (5.0%) | 664 (3.7%) | 0.07 |
| Insurance level |  |  |  |  |  |  |  |  |  |  |  |  |  |  |  |
| 1~2 | - | - |  | 11716 (13.8%) | 17450 (14.1%) | 0.01 | 9405 (14.8%) | 13586 (15.39%) | 0.02 | 3284 (11.6%) | 4416 (11.3%) | 0.01 | 2244 (17.5%) | 3410 (19.0%) | 0.04 |
| 3~4 | - | - |  | 11058 (13.0%) | 16780 (13.5%) | 0.02 | 8293 (13.1%) | 13223 (14.98%) | 0.06 | 2618 (9.2%) | 3817 (9.8%) | 0.02 | 2116 (16.5%) | 3382 (18.9%) | 0.06 |
| 5~6 | - | - |  | 13010 (15.3%) | 20213 (16.3%) | 0.03 | 9685 (15.2%) | 13898 (15.75%) | 0.01 | 3332 (11.7%) | 4865 (12.4%) | 0.02 | 2445 (19.1%) | 3434 (19.2%) | 0.00 |
| 7~8 | - | - |  | 17825 (20.9%) | 26733 (21.6%) | 0.02 | 12900 (20.3%) | 18033 (20.43%) | 0.00 | 5573 (19.6%) | 8064 (20.6%) | 0.03 | 2116 (16.5%) | 3454 (19.3%) | 0.07 |
| 9~10 | - | - |  | 27082 (31.8%) | 36341 (29.3%) | 0.05 | 20245 (31.9%) | 25515 (28.91%) | 0.06 | 12372 (43.4%) | 15872 (40.5%) | 0.06 | 2854 (22.3%) | 3486 (19.4%) | 0.07 |
| 0 | - | - |  | 4433 (5.2%) | 6413 (5.2%) | 0.00 | 3005 (4.7%) | 3995 (4.53%) | 0.01 | 1311 (4.6%) | 2120 (5.4%) | 0.04 | 821 (6.4%) | 764 (4.3%) | 0.10 |
| CCI index |  |  |  |  |  |  |  |  |  |  |  |  |  |  |  |
| 0–1 | 20289 (17.2%) | 18678 (9.9%) | 0.22 | 8715 (10.2%) | 10853 (8.8%) | 0.05 | 13873 (21.8%) | 13121 (14.87%) | 0.18 | 2196 (7.7%) | 1592 (4.1%) | 0.16 | 3211 (25.1%) | 3264 (18.2%) | 0.17 |
| 2 | 22768 (19.3%) | 29278 (15.5%) | 0.10 | 14446 (17.0%) | 14731 (11.9%) | 0.15 | 15422 (24.3%) | 17094 (19.37%) | 0.12 | 2913 (10.2%) | 2474 (6.3%) | 0.14 | 2863 (22.4%) | 3317 (18.5%) | 0.10 |
| 3 | 19999 (16.9%) | 37161 (19.6%) | 0.07 | 12972 (15.2%) | 21154 (17.1%) | 0.05 | 12100 (19.1%) | 19618 (22.23%) | 0.08 | 3247 (11.4%) | 4134 (10.6%) | 0.03 | 2116 (16.5%) | 3541 (19.8%) | 0.08 |
| 4+ | 54983 (46.6%) | 104341 (55.1%) | 0.17 | 48991 (57.6%) | 77192 (62.3%) | 0.10 | 22138 (34.8%) | 38417 (43.53%) | 0.18 | 20134 (70.7%) | 30954 (79.1%) | 0.19 | 4622 (36.1%) | 7808 (43.6%) | 0.15 |
| SEER summary stage |  |  |  |  |  |  |  |  |  |  |  |  |  |  |  |
| Localized | 49300 (41.8%) | 77871 (41.1%) | 0.01 | 60196 (70.7%) | 88604 (71.5%) | 0.02 | 38449 (60.5%) | 54016 (61.21%) | 0.01 | 20223 (71.0%) | 27890 (71.2%) | 0.01 | 8453 (66.0%) | 12093 (67.5%) | 0.03 |
| Regional | 68739 (58.2%) | 111587 (58.9%) |  | 24928 (29.3%) | 35326 (28.5%) |  | 25084 (39.5%) | 34234 (38.79%) |  | 8267 (29.0%) | 11264 (28.8%) |  | 4359 (34.0%) | 5837 (32.6%) |  |
| BMD test | 18061 (15.3%) | 23869 (12.6%) | 0.08 | 2243 (2.6%) | 3833 (3.1%) | 0.03 | 25113 (39.5%) | 49943 (56.59%) | 0.34 | 3223 (11.3%) | 5084 (13.0%) | 0.05 | 1771 (13.8%) | 3126 (17.4%) | 0.10 |
| Osteoporotic drug | - | - |  | 413 (0.5%) | 885 (0.7%) | 0.03 | 2046 (3.2%) | 3378 (3.83%) | 0.03 | 274 (1.0%) | 547 (1.4%) | 0.04 | 306 (2.4%) | 557 (3.1%) | 0.04 |

## Supplementary Table 6. Comparison of baseline characteristics according to the treatment in thyroid cancer.

|  | DB_III_ | | DB_DII_ | | Effect Size |
| --- | --- | --- | --- | --- | --- |
| Variable | No Vitamin D | Vitamin D | No Vitamin D | Vitamin D |  |
| Sex |  |  |  |  |  |
| Male | 17940 (16.42%) | 1179 (13.41%) | 32147 (19.21%) | 3475 (15.69%) | 0.07 |
| Female | 91307 (83.58%) | 7613 (86.59%) | 135158 (80.76%) | 18678 (84.31%) |  |
| Age at diagnosis (year) |  |  |  |  |  |
| <40 | 22375 (20.48%) | 1467 (16.69%) | 38219 (22.84%) | 4447 (20.07%) | 0.06 |
| 40–49 | 35672 (32.65%) | 2779 (31.61%) | 50344 (30.09%) | 6483 (29.26%) | 0.06 |
| 50–59 | 34834 (31.89%) | 3091 (35.16%) | 49708 (29.71%) | 6921 (31.24%) | 0.05 |
| ≥60 | 16366 (14.98%) | 1455 (16.55%) | 29034 (17.36%) | 4302 (19.42%) | 0.07 |
| Calendar year at diagnosis |  |  |  |  |  |
| 2008–2010 | 35699 (32.68%) | 2329 (26.49%) | 55849 (33.38%) | 5777 (26.08%) | 0.02 |
| 2011–2013 | 49285 (45.11%) | 4574 (52.02%) | 75551 (45.16%) | 10952 (49.44%) | 0.00 |
| 2014–2016 | 24263 (22.21%) | 1889 (21.49%) | 35905 (21.46%) | 5424 (24.48%) | 0.02 |
| Region |  |  |  |  |  |
| Urban | 82107 (75.16%) | 6654 (75.68%) | 119999 (71.72%) | 16099 (72.67%) | 0.08 |
| Rural | 27140 (24.84%) | 2138 (24.32%) | 47306 (28.28%) | 6054 (27.33%) |  |
| Insurance type |  |  |  |  |  |
| Local | 32571 (29.81%) | 2682 (30.51%) | 62187 (37.17%) | 8629 (38.95%) | 0.16 |
| Employment | 74711 (68.39%) | 5938 (67.54%) | 102136 (61.05%) | 13112 (59.19%) | 0.15 |
| Medical aids | 1965 (1.8%) | 172 (1.96%) | 2982 (1.78%) | 412 (1.86%) | 0.00 |
| CCI index |  |  |  |  |  |
| 0–1 | 18846 (17.25%) | 1443 (16.41%) | 16293 (9.74%) | 2385 (10.77%) | 0.22 |
| 2 | 21120 (19.33%) | 1648 (18.74%) | 26047 (15.57%) | 3231 (14.58%) | 0.10 |
| 3 | 18513 (16.95%) | 1486 (16.9%) | 33065 (19.76%) | 4096 (18.49%) | 0.07 |
| 4+ | 50768 (46.47%) | 4215 (47.94%) | 91900 (54.93%) | 12441 (56.16%) | 0.17 |
| SEER summary stage |  |  |  |  |  |
| Localized | 45977 (42.09%) | 3323 (37.8%) | 70140 (41.92%) | 7731 (34.9%) | 0.00 |
| Regional | 63270 (57.91%) | 5469 (62.2%) | 97165 (58.08%) | 14422 (65.1%) | 0.00 |
| BMD test | 16595 (15.19%) | 1466 (16.67%) | 22190 (13.26%) | 2750 (12.41%) | 0.06 |
| Osteoporotic drug | 3147 (2.88%) |  | 5828 (3.48%) | 902 (4.07%) | 0.03 |

Abbreviation: CCI, Charlson Comorbidity Index; BMD, bone mass density.

## Supplementary Table 7. Comparison of baseline characteristics according to the treatment in gastric cancer.

|  | DB_III_ | |  | DB_DII_ | |  | Effect Size |
| --- | --- | --- | --- | --- | --- | --- | --- |
| Variable | Total Gastrectomy | Subtotal Gastrectomy | ESD/EMR | Total Gastrectomy | Subtotal Gastrectomy | ESD/EMR |  |
| Sex |  |  |  |  |  |  |  |
| Male | 10736 (74.41%) | 38196 (72.65%) | 15173 (83.72%) | 15608 (74.54%) | 55606 (72.21%) | 21426 (82.45%) | 0.00 |
| Female | 3692 (25.59%) | 14376 (27.35%) | 2951 (16.28%) | 5332 (25.46%) | 21396 (27.79%) | 4562 (17.55%) |  |
| Age at diagnosis (year) |  |  |  |  |  |  |  |
| <40 | 757 (5.25%) | 2399 (4.56%) | 143 (0.79%) | 1171 (5.59%) | 3944 (5.12%) | 272 (1.05%) | 0.02 |
| 40–49 | 2392 (16.58%) | 8673 (16.5%) | 1336 (7.37%) | 3499 (16.71%) | 12995 (16.88%) | 2177 (8.38%) | 0.00 |
| 50–59 | 4411 (30.57%) | 16734 (31.83%) | 5446 (30.05%) | 6070 (28.99%) | 22422 (29.12%) | 7106 (27.34%) | 0.04 |
| 60–69 | 4248 (29.44%) | 15424 (29.34%) | 6836 (37.72%) | 5972 (28.52%) | 21598 (28.05%) | 9112 (35.06%) | 0.02 |
| 70–79 | 2432 (16.86%) | 8470 (16.11%) | 3966 (21.88%) | 3790 (18.1%) | 13891 (18.04%) | 6201 (23.86%) | 0.03 |
| ≥80 | 188 (1.3%) | 872 (1.66%) | 397 (2.19%) | 438 (2.09%) | 2152 (2.79%) | 1120 (4.31%) | 0.06 |
| Calendar year at diagnosis |  |  |  |  |  |  |  |
| 2008–2010 | 5639 (39.08%) | 19838 (37.73%) | 1583 (8.73%) | 8229 (39.3%) | 29555 (38.38%) | 2504 (9.64%) | 0.01 |
| 2011–2013 | 4938 (34.23%) | 18117 (34.46%) | 7608 (41.98%) | 7171 (34.25%) | 26264 (34.11%) | 10861 (41.79%) | 0.00 |
| 2014–2016 | 3851 (26.69%) | 14617 (27.8%) | 8933 (49.29%) | 5540 (26.46%) | 21183 (27.51%) | 12623 (48.57%) | 0.01 |
| Region |  |  |  |  |  |  |  |
| Urban | 9779 (67.78%) | 35808 (68.11%) | 12751 (70.35%) | 13821 (66%) | 50920 (66.13%) | 17651 (67.92%) | 0.04 |
| Rural | 4649 (32.22%) | 16764 (31.89%) | 5373 (29.65%) | 7119 (34%) | 26082 (33.87%) | 8337 (32.08%) |  |
| Insurance type |  |  |  |  |  |  |  |
| Local | 5080 (35.21%) | 17596 (33.47%) | 5572 (30.74%) | 7162 (34.22%) | 25632 (33.3%) | 7648 (29.44%) | 0.02 |
| Employment | 8791 (60.93%) | 32821 (62.43%) | 12074 (66.62%) | 13164 (62.9%) | 48906 (63.54%) | 17715 (68.2%) | 0.04 |
| Medical aids | 557 (3.86%) | 2155 (4.1%) | 478 (2.64%) | 603 (2.88%) | 2435 (3.16%) | 612 (2.36%) | 0.05 |
| Insurance level |  |  |  |  |  |  |  |
| 1~2 | 2032 (14.08%) | 7358 (14%) | 2326 (12.83%) | 3052 (14.57%) | 11092 (14.4%) | 3306 (12.72%) | 0.01 |
| 3~4 | 1918 (13.29%) | 7004 (13.32%) | 2136 (11.79%) | 2978 (14.22%) | 10613 (13.78%) | 3189 (12.27%) | 0.03 |
| 5~6 | 2303 (15.96%) | 8189 (15.58%) | 2518 (13.89%) | 3542 (16.91%) | 12810 (16.64%) | 3861 (14.86%) | 0.03 |
| 7~8 | 3156 (21.87%) | 10904 (20.74%) | 3765 (20.77%) | 4606 (22%) | 16566 (21.51%) | 5561 (21.4%) | 0.00 |
| 9~10 | 4247 (29.44%) | 16197 (30.81%) | 6638 (36.63%) | 5719 (27.31%) | 21767 (28.27%) | 8855 (34.07%) | 0.05 |
| 0 | 772 (5.35%) | 2920 (5.55%) | 741 (4.09%) | 1043 (4.98%) | 4154 (5.39%) | 1216 (4.68%) | 0.02 |
| CCI index |  |  |  |  |  |  |  |
| 0–1 | 1467 (10.17%) | 5708 (10.86%) | 1540 (8.5%) | 2105 (10.05%) | 7210 (9.36%) | 1538 (5.92%) | 0.00 |
| 2 | 2351 (16.29%) | 9174 (17.45%) | 2921 (16.12%) | 2566 (12.25%) | 9534 (12.38%) | 2631 (10.12%) | 0.12 |
| 3 | 2162 (15.61%) | 8206 (15.61%) | 2604 (14.37%) | 3746 (17.89%) | 13394 (17.39%) | 4014 (15.45%) | 0.06 |
| 4+ | 8448 (56.08%) | 29484 (56.08%) | 11059 (61.02%) | 12523 (59.8%) | 46864 (60.86%) | 17805 (68.51%) | 0.08 |
| SEER summary stage |  |  |  |  |  |  |  |
| Localized | 6431 (44.57%) | 35850 (68.19%) | 17915 (98.85%) | 9800 (46.8%) | 53084 (68.94%) | 25720 (98.97%) | 0.05 |
| Regional | 7997 (55.43%) | 16722 (31.81%) | 209 (1.15%) | 11140 (53.2%) | 23918 (31.06%) | 268 (1.03%) |  |
| BMD test | 348 (2.41%) | 1415 (2.69%) | 480 (2.65%) | 574 (2.74%) | 2483 (3.22%) | 776 (2.99%) | 0.02 |
| Osteoporotic drug | 68 (0.47%) | 238 (0.45%) | 107 (0.59%) | 117 (0.56%) | 552 (0.72%) | 216 (0.83%) | 0.01 |

Abbreviation: CCI, Charlson Comorbidity Index; BMD, bone mass density.

## Supplementary Table 8. Comparison of baseline characteristics according to the treatment in breast cancer.

|  | DB_III_ | |  |  | DB_DII_ | |  |  | Effect Size |
| --- | --- | --- | --- | --- | --- | --- | --- | --- | --- |
| Variable | None | AI-only | TAM-only | AI+TAM | None | AI-only | TAM-only | AI+TAM |  |
| Age at diagnosis (year) |  |  |  |  |  |  |  |  |  |
| <40 | 3183 (15.77%) | 13 (0.09%) | 5463 (18.7%) | 1 (0.28%) | 4370 (17.26%) | 20 (0.1%) | 7848 (18.45%) | 4 (0.59%) | 0.04 |
| 40–49 | 7269 (36%) | 1118 (8.12%) | 18431 (63.07%) | 80 (22.16%) | 8425 (33.27%) | 1268 (6.43%) | 26076 (61.3%) | 143 (21.15%) | 0.06 |
| 50–59 | 6898 (34.17%) | 7983 (58%) | 4320 (14.78%) | 192 (53.19%) | 8539 (33.72%) | 10782 (54.7%) | 6935 (16.3%) | 349 (51.63%) | 0.01 |
| 60–69 | 2175 (10.78%) | 3661 (26.6%) | 690 (2.36%) | 66 (18.28%) | 2912 (11.5%) | 5690 (28.87%) | 1061 (2.49%) | 134 (19.82%) | 0.02 |
| ≥70 | 663 (3.28%) | 989 (7.19%) | 316 (1.08%) | 22 (6.09%) | 1079 (4.26%) | 1951 (9.9%) | 618 (1.45%) | 46 (6.8%) | 0.05 |
| Calendar year at diagnosis |  |  |  |  |  |  |  |  |  |
| 2008–2010 | 6366 (31.53%) | 3977 (28.89%) | 9019 (30.87%) | 124 (34.35%) | 8617 (34.03%) | 5595 (28.39%) | 12673 (29.79%) | 235 (34.76%) | 0.05 |
| 2011–2013 | 6230 (30.86%) | 5026 (36.52%) | 10627 (36.37%) | 130 (36.01%) | 8472 (33.45%) | 6855 (34.78%) | 14605 (34.33%) | 232 (34.32%) | 0.06 |
| 2014–2016 | 7592 (37.61%) | 4761 (34.59%) | 9574 (32.76%) | 107 (29.64%) | 8236 (32.52%) | 7261 (36.84%) | 15260 (35.87%) | 209 (30.92%) | 0.11 |
| Region |  |  |  |  |  |  |  |  |  |
| Urban | 15480 (76.68%) | 10553 (76.67%) | 22637 (77.47%) | 301 (83.38%) | 19036 (75.17%) | 14779 (74.98%) | 32099 (75.46%) | 539 (79.73%) | 0.04 |
| Rural | 4708 (23.32%) | 3211 (23.33%) | 6583 (22.53%) | 60 (16.62%) | 6289 (24.83%) | 4932 (25.02%) | 10439 (24.54%) | 137 (20.27%) |  |
| Insurance type |  |  |  |  |  |  |  |  |  |
| Local | 7030 (34.82%) | 4830 (35.09%) | 9621 (32.92%) | 139 (38.5%) | 9069 (35.81%) | 6971 (35.37%) | 14139 (33.24%) | 251 (37.13%) | 0.02 |
| Employment | 12480 (61.82%) | 8385 (60.92%) | 18888 (64.64%) | 213 (59%) | 15626 (61.7%) | 12139 (61.58%) | 27598 (64.88%) | 412 (60.95%) | 0.00 |
| Medical aids | 678 (3.36%) | 549 (3.99%) | 711 (2.44%) | 9 (2.49%) | 615 (2.43%) | 594 (3.01%) | 782 (1.84%) | 12 (1.78%) | 0.06 |
| Insurance level |  |  |  |  |  |  |  |  |  |
| 1~2 | 3082 (15.27%) | 2016 (14.65%) | 4253 (14.55%) | 54 (14.96%) | 3990 (15.76%) | 3055 (15.5%) | 6430 (15.12%) | 111 (16.42%) | 0.01 |
| 3~4 | 2730 (13.52%) | 1774 (12.89%) | 3737 (12.79%) | 52 (14.4%) | 3962 (15.64%) | 2808 (14.25%) | 6332 (14.89%) | 121 (17.9%) | 0.06 |
| 5~6 | 3210 (15.9%) | 2100 (15.26%) | 4333 (14.83%) | 42 (11.63%) | 4212 (16.63%) | 3094 (15.7%) | 6501 (15.28%) | 91 (13.46%) | 0.02 |
| 7~8 | 4147 (20.54%) | 2795 (20.31%) | 5874 (20.1%) | 84 (23.27%) | 5222 (20.62%) | 3965 (20.12%) | 8733 (20.53%) | 113 (16.72%) | 0.00 |
| 9~10 | 5988 (29.66%) | 4322 (31.4%) | 9818 (33.6%) | 117 (32.41%) | 6742 (26.62%) | 5782 (29.33%) | 12777 (30.04%) | 214 (31.66%) | 0.07 |
| 0 | 1031 (5.11%) | 757 (5.5%) | 1205 (4.13%) | 12 (3.32%) | 1197 (4.73%) | 1007 (5.11%) | 1765 (4.15%) | 26 (3.85%) | 0.02 |
| CCI index |  |  |  |  |  |  |  |  |  |
| 0–1 | 4438 (21.98%) | 2018 (14.66%) | 7371 (25.23%) | 46 (12.74%) | 3778 (14.92%) | 1837 (9.32%) | 7453 (17.52%) | 53 (7.84%) | 0.18 |
| 2 | 4694 (23.25%) | 2489 (18.08%) | 8176 (27.98%) | 63 (17.45%) | 4671 (18.44%) | 2533 (12.85%) | 9817 (23.08%) | 73 (10.8%) | 0.12 |
| 3 | 3811 (18.88%) | 2394 (17.39%) | 5824 (19.93%) | 71 (19.67%) | 5518 (21.79%) | 3614 (18.33%) | 10366 (24.37%) | 120 (17.75%) | 0.07 |
| 4+ | 7245 (35.89%) | 6863 (49.86%) | 7849 (26.86%) | 181 (50.14%) | 11358 (44.85%) | 11727 (59.49%) | 14902 (35.03%) | 430 (63.61%) | 0.18 |
| SEER summary stage |  |  |  |  |  |  |  |  |  |
| Localized | 11555 (57.24%) | 8404 (61.06%) | 18230 (62.39%) | 260 (72.02%) | 15033 (59.36%) | 12011 (60.94%) | 26498 (62.29%) | 474 (70.12%) | 0.04 |
| Regional | 8633 (42.76%) | 5360 (38.94%) | 10990 (37.61%) | 101 (27.98%) | 10292 (40.64%) | 7700 (39.06%) | 16040 (37.71%) | 202 (29.88%) | 0.04 |
| BMD test | 6118 (30.31%) | 8825 (64.12%) | 9939 (34.01%) | 231 (63.99%) | 11805 (46.61%) | 16055 (81.45%) | 21584 (50.74%) | 499 (73.82%) | 0.34 |
| Osteoporotic drug | 483 (2.39%) | 1018 (7.4%) | 523 (1.79%) | 22 (6.09%) | 725 (2.86%) | 1723 (8.74%) | 881 (2.07%) | 49 (7.25%) | 0.03 |

Abbreviation: CCI, Charlson Comorbidity Index; BMD, bone mass density.

## Supplementary Table 9. Comparison of baseline characteristics according to the treatment in prostate cancer.

|  | DB_III_ | | DB_DII_ | | Effect Size |
| --- | --- | --- | --- | --- | --- |
| Variable | No ADT | ADT | No ADT | ADT |  |
| Age at diagnosis (year) |  |  |  |  |  |
| 40–49 | 236 (1.41%) | 55 (0.47%) | 301 (1.44%) | 75 (0.41%) | 0.00 |
| 50–59 | 2933 (17.46%) | 930 (7.95%) | 3265 (15.64%) | 1225 (6.7%) | 0.05 |
| 60–69 | 8269 (49.24%) | 3635 (31.08%) | 9651 (46.22%) | 5047 (27.62%) | 0.06 |
| 70–79 | 5105 (30.4%) | 5675 (48.52%) | 7140 (34.19%) | 8830 (48.32%) | 0.08 |
| ≥80 | 251 (1.49%) | 1401 (11.98%) | 524 (2.51%) | 3096 (16.94%) | 0.07 |
| Calendar year at diagnosis |  |  |  |  |  |
| 2008–2010 | 4594 (27.36%) | 3646 (31.17%) | 6218 (29.78%) | 6058 (33.15%) | 0.05 |
| 2011–2013 | 5974 (35.57%) | 4136 (35.36%) | 7650 (36.64%) | 6309 (34.53%) | 0.02 |
| 2014–2016 | 6226 (37.07%) | 3914 (33.46%) | 7013 (33.59%) | 5906 (32.32%) | 0.07 |
| Region |  |  |  |  |  |
| Urban | 12373 (73.68%) | 8022 (68.59%) | 15186 (72.73%) | 12164 (66.57%) | 0.02 |
| Rural | 4421 (26.32%) | 3674 (31.41%) | 5695 (27.27%) | 6109 (33.43%) | 0.02 |
| Insurance type |  |  |  |  |  |
| Local | 5255 (31.29%) | 3358 (28.71%) | 6231 (29.84%) | 5317 (29.1%) | 0.03 |
| Employment | 11239 (66.92%) | 7727 (66.07%) | 14228 (68.14%) | 12137 (66.42%) | 0.03 |
| Medical aids | 300 (1.79%) | 611 (5.22%) | 341 (1.63%) | 810 (4.43%) | 0.01 |
| Insurance level |  |  |  |  |  |
| 1~2 | 1853 (11.02%) | 1431 (12.23%) | 2236 (10.71%) | 2180 (11.93%) | 0.01 |
| 3~4 | 1498 (8.94%) | 1120 (9.58%) | 2057 (9.85%) | 1760 (9.63%) | 0.03 |
| 5~6 | 1948 (11.6%) | 1384 (11.83%) | 2557 (12.25%) | 2308 (12.63%) | 0.02 |
| 7~8 | 3257 (19.39%) | 2316 (19.8%) | 4322 (20.7%) | 3742 (20.48%) | 0.03 |
| 9~10 | 7724 (45.99%) | 4648 (39.74%) | 8847 (42.37%) | 7025 (38.44%) | 0.07 |
| 0 | 514 (3.08%) | 797 (6.81%) | 862 (4.13%) | 1258 (6.88%) | 0.06 |
| CCI index |  |  |  |  |  |
| 0–1 | 1326 (7.9%) | 870 (7.44%) | 911 (4.36%) | 681 (3.73%) | 0.15 |
| 2 | 1877 (11.18%) | 1036 (8.86%) | 1466 (7.02%) | 1008 (5.52%) | 0.15 |
| 3 | 2080 (12.39%) | 1167 (9.98%) | 2416 (11.57%) | 1718 (9.4%) | 0.03 |
| 4+ | 11511 (68.54%) | 8623 (73.73%) | 16088 (77.05%) | 14866 (81.36%) | 0.19 |
| SEER summary stage |  |  |  |  |  |
| Localized | 12992 (77.36%) | 7231 (61.82%) | 16233 (77.74%) | 11657 (63.79%) | 0.01 |
| Regional | 3802 (22.64%) | 4465 (38.18%) | 4648 (22.26%) | 6616 (36.21%) |  |
| BMD test | 1452 (8.65%) | 1771 (15.14%) | 2183 (10.45%) | 2901 (15.88%) | 0.06 |
| Osteoporotic drug | 108 (0.64%) | 166 (1.42%) | 207 (0.99%) | 340 (1.86%) | 0.04 |

Abbreviation: CCI, Charlson Comorbidity Index; BMD, bone mass density; ADT, androgen deprivation therapy.

## Supplementary Table 10. Comparison of baseline characteristics according to the treatment in cervical cancer.

|  | DB_III_ | | DB_DII_ | | Effect Size |
| --- | --- | --- | --- | --- | --- |
| Variable | No RT | RT | No RT | RT |  |
| Age at diagnosis (year) |  |  |  |  |  |
| 40–49 | 2388 (23.74%) | 455 (16.53%) | 3427 (24.08%) | 610 (16.5%) | 0.00 |
| 50–59 | 3386 (33.66%) | 920 (33.42%) | 4591 (32.25%) | 1136 (30.74%) | 0.06 |
| 60–69 | 2446 (24.32%) | 804 (29.2%) | 3311 (23.26%) | 1082 (29.27%) | 0.00 |
| 70–79 | 1118 (11.11%) | 359 (13.04%) | 1695 (11.91%) | 506 (13.69%) | 0.02 |
| ≥80 | 721 (7.17%) | 215 (7.81%) | 1210 (8.51%) | 362 (9.79%) | 0.07 |
| Calendar year at diagnosis |  |  |  |  |  |
| 2008–2010 | 4642 (46.15%) | 221 (8.03%) | 6631 (46.59%) | 317 (8.58%) | 0.02 |
| 2011–2013 | 2960 (29.43%) | 1290 (46.86%) | 4139 (29.08%) | 1750 (47.35%) | 0.01 |
| 2014–2016 | 2457 (24.43%) | 1242 (45.11%) | 3464 (24.34%) | 1629 (44.07%) | 0.02 |
| Region |  |  |  |  |  |
| Urban | 7305 (72.62%) | 1979 (71.89%) | 10053 (70.63%) | 2591 (70.1%) | 0.04 |
| Rural | 2754 (27.38%) | 774 (28.11%) | 4181 (29.37%) | 1105 (29.9%) |  |
| Insurance type |  |  |  |  |  |
| Local | 4030 (40.06%) | 1164 (42.28%) | 5818 (40.87%) | 1562 (42.26%) | 0.00 |
| Employment | 5520 (54.88%) | 1455 (52.85%) | 7877 (55.34%) | 2009 (54.36%) | 0.03 |
| Medical aids | 509 (5.06%) | 134 (4.87%) | 539 (3.79%) | 125 (3.38%) | 0.08 |
| Insurance level |  |  |  |  |  |
| 1~2 | 1784 (17.74%) | 460 (16.71%) | 2719 (19.1%) | 691 (18.7%) | 0.05 |
| 3~4 | 1624 (16.14%) | 492 (17.87%) | 2644 (18.58%) | 738 (19.97%) | 0.05 |
| 5~6 | 1827 (18.16%) | 505 (18.34%) | 2725 (19.14%) | 709 (19.18%) | 0.02 |
| 7~8 | 1924 (19.13%) | 521 (18.92%) | 2722 (19.12%) | 732 (19.81%) | 0.02 |
| 9~10 | 2255 (22.42%) | 599 (21.76%) | 2794 (19.63%) | 692 (18.72%) | 0.08 |
| 0 | 645 (6.41%) | 176 (6.39%) | 630 (4.43%) | 134 (3.63%) | 0.13 |
| CCI index |  |  |  |  |  |
| 0–1 | 2533 (25.18%) | 678 (24.63%) | 2553 (17.94%) | 711 (19.24%) | 0.13 |
| 2 | 2243 (22.3%) | 620 (22.52%) | 2640 (18.55%) | 677 (18.32%) | 0.10 |
| 3 | 1654 (16.44%) | 462 (16.78%) | 2850 (20.02%) | 691 (18.7%) | 0.05 |
| 4+ | 3629 (36.08%) | 993 (36.07%) | 6191 (43.49%) | 1617 (43.75%) | 0.16 |
| SEER summary stage |  |  |  |  |  |
| Localized | 7337(72.94%) | 1116(40.54%) | 10595 (74.43%) | 1498 (40.53%) | 0.00 |
| Regional | 2722(27.06%) | 1637(59.46%) | 3639 (25.57%) | 2198 (59.47%) |  |
| BMD test | 1332(13.24%) | 439(15.95%) | 2356 (16.55%) | 770 (20.83%) | 0.13 |
| Osteoporotic drug | 216(2.15%) | 90(3.27%) | 408 (2.87%) | 149 (4.03%) | 0.04 |

Abbreviation: CCI, Charlson Comorbidity Index; BMD, bone mass density; RT, radiation therapy.

## Supplementary Table 11. Incidence rate estimation of osteoporotic fracture per cancer type.

| Variable | Thyroid Cancer | | | Gastric Cancer | | | Breast Cancer | | | Prostate Cancer | | | Cervical Cancer | | |
| --- | --- | --- | --- | --- | --- | --- | --- | --- | --- | --- | --- | --- | --- | --- | --- |
|  | DB_III_ | DB_DII_ | Effect Size | DB_III_ | DB_DII_ | Effect Size | DB_III_ | DB_DII_ | Effect Size | DB_III_ | DB_DII_ | Effect Size | DB_III_ | DB_DII_ | Effect Size |
| Osteoporotic fracture* | |  |  |  |  |  |  |  |  |  |  |  |  |  |  |
| Group1 | 1.3 (1.2-1.3) | 1.5 (1.5-1.6) | 0.02 | 2.6 (2.4-2.9) | 3.2 (3.0-3.4) | 0.02 | 2.2 (2.1-2.4) | 2.2 (2.1-2.4) | 0.00 | 1.3 (1.2-1.4) | 1.9 (1.8-2.1) | 0.05 | 0.4 (0.3-0.5) | 0.5 (0.4-0.6) | 0.01 |
| Group2 | 1.1 (0.9-1.2) | 1.4 (1.3-1.5) | 0.00 | 2.1 (2.0-2.2) | 2.7 (2.6-2.8) | 0.04 | 3.5 (3.2-3.7) | 3.6 (3.4-3.8) | 0.00 | 4.1 (3.8-4.5) | 5.4 (5.1-5.7) | 0.04 | 1.1 (0.8-1.6) | 1.0 (0.7-1.3) | 0.01 |
| Group3 | - | - | - | 1.8 (1.6-1.9) | 2.3 (2.2-2.5) | 0.02 | 1.2 (1.1-1.3) | 1.2 (1.1-1.3) | 0.00 | - | - | - | - | - | - |
| Group4 | - | - | - | - | - | - | 1.3 (0.6-2.4) | 2.9 (1.9-3.9) | 0.01 | - | - | - | - | - | - |
| Hip fracture |  |  |  |  |  |  |  |  |  |  |  |  |  |  |  |
| Group1 | 0.1 (0.0-0.1) | 0.1 (0.1-0.1) | 0.09 | 0.6 (0.5-0.7) | 0.6 (0.5-0.7) | 0.00 | 0.2 (0.2-0.3) | 0.2 (0.2-0.3) | 0.00 | 0.3 (0.2-0.3) | 0.6 (0.5-0.7) | 0.04 | - | - | - |
| Group2 | 0.0 (0.0-0.1) | 0.1 (0.0-0.1) | 0.01 | 0.4 (0.4-0.5) | 0.5 (0.4-0.5) | 0.01 | 0.2 (0.1-0.2) | 0.2 (0.2-0.3) | 0.00 | 1.0 (0.9-1.2) | 2.0 (1.8-2.2) | 0.06 | - | - | - |
| Group3 | - | - | - | 0.3 (0.2-0.4) | 0.4 (0.3-0.4) | 0.01 | 0.1 (0.0-0.1) | 0.1 (0.1-0.1) | 0.00 | - | - | - | - | - | - |
| Group4 | - | - | - | - | - | - | - | 0.1 (0.0-0.3) | - | - | - | - | - | - | - |
| Vertebral fracture |  |  |  |  |  |  |  |  |  |  |  |  |  |  |  |
| Group1 | 0.3 (0.3-0.4) | 0.6 (0.6-0.7) | 0.03 | 0.9 (0.1-1.1) | 1.5 (1.4-1.7) | 0.01 | 0.8 (0.7-0.9) | 0.8 (0.7-0.9) | 0.00 | 0.5 (0.5-0.6) | 1.2 (1.0-1.3) | 0.06 | - | - | - |
| Group2 | 0.2 (0.2-0.3) | 0.6 (0.5-0.7) | 0.01 | 0.8 (0.8-0.9) | 1.4 (1.3-1.5) | 0.04 | 1.1 (1.0-1.3) | 1.3 (1.2-1.4) | 0.01 | 2.1 (1.8-2.3) | 3.3 (3.0-3.5) | 0.05 | - | - | - |
| Group3 | - | - | - | 0.7 (0.6-0.8) | 1.2 (1.1-1.4) | 0.02 | 0.4 (0.3-0.4) | 0.4 (0.3-0.4) | 0.00 | - | - | - | - | - | - |
| Group4 | - | - | - | - | - | - | 0.3 (0.0-0.9) | 1.0 (0.5-1.7) | 0.01 | - | - | - | - | - | - |
| Distal radius fracture |  |  |  |  |  |  |  |  |  |  |  |  |  |  |  |
| Group1 | 0.9 (0.8-0.9) | 0.9 (0.8-0.9) | 0.00 | 1.2 (1.0-1.1) | 1.1 (1.0-1.3) | 0.01 | 1.3 (1.1-1.4) | 1.2 (1.1-1.3) | 0.01 | 0.5 (0.4-0.6) | 0.5 (0.4-0.6) | 0.00 | - | - | - |
| Group2 | 0.8 (0.7-0.9) | 0.8 (0.7-0.9) | 0.00 | 0.9 (0.9-1.0) | 1.0 (0.9-1.0) | 0.01 | 2.3 (2.1-2.5) | 2.2 (2.0-2.3) | 0.00 | 1.2 (1-1.3) | 1.2 (1.0-1.3) | 0.00 | - | - | - |
| Group3 | - | - | - | 0.8 (0.7-0.9) | 0.8 (0.7-0.9) | 0.00 | 0.7 (0.6-0.8) | 0.7 (0.7-0.8) | 0.00 | - | - | - | - | - | - |
| Group4 | - | - | - | - | - | - | 1.0 (0.4-1.9) | 1.8 (1.1-2.7) | 0.01 | - | - | - | - | - | - |
| Proximal humerus fracture |  |  |  |  |  |  |  |  |  |  |  |  |  |  |  |
| Group1 | 0.03 (0.02-0.04) | 0.04 (0.03-0.05) | 0.00 | 0.1 (0.1-0.2) | 0.1 (0.1-0.1) | 0.00 | 0.1 (0.0-0.1) | 0.1 (0.0-0.1) | 0.02 | 0.0 (0.0-0.1) | 0.1 (0.0-0.1) | 0.02 | - | - | - |
| Group2 | 0.02 (0.00-0.05) | 0.03 (0.01-0.04) | 0.00 | 0.1 (0.1-0.1) | 0.1 (0.1-0.1) | 0.00 | 0.1 (0.1-0.2) | 0.1 (0.1-0.2) | 0.00 | 0.1 (0.1-0.1) | 0.1 (0.1-0.2) | 0.00 | - | - | - |
| Group3 | - | - | - | 0.1 (0.1-0.1) | 0.1 (0.0-0.1) | 0.00 | 0.1 (0.0-0.1) | 0.1 (0.1-0.1) | 0.05 | - | - | - | - | - | - |
| Group4 | - | - | - | - | - | - | - | 0.2 (0.0-0.5) | - | - | - | - | - | - | - |

Values were reported as incidence rates with 95% confidence intervals.

## Supplementary Table 12. Multiple Cox proportional hazards regression analysis for osteoporotic fractures in patients with cancer.

| Variable | Thyroid Cancer | | | Gastric Cancer | | | Breast Cancer | | | Prostate Cancer | | | Cervical Cancer | | |
| --- | --- | --- | --- | --- | --- | --- | --- | --- | --- | --- | --- | --- | --- | --- | --- |
|  | DB_III_ | DB_DII_ | Effect Size | DB_III_ | DB_DII_ | Effect Size | DB_III_ | DB_DII_ | Effect Size | DB_III_ | DB_DII_ | Effect Size | DB_III_ | DB_DII_ | Effect Size |
| Treatment |  |  |  |  |  |  |  |  |  |  |  |  |  |  |  |
| Group1 | 1 | 1 |  | 1 | 1 |  | 1 | 1 |  | 1 | 1 |  | 1 | 1 |  |
| Group2 | 0.83 (0.70-0.99) | 0.83 (0.75-0.91) | 0.00 | 1.24 (1.10-1.39) | 1.26 (1.15-1.37) | 0.00 | 0.95 (0.85-1.07) | 1.03 (0.94-1.14) | 0.01 | 2.14 (1.85-2.48) | 1.96 (1.76-2.17) | 0.01 | 2.62 (1.63-4.23) | 1.80 (1.18-2.73) | 0.01 |
| Group3 |  |  |  |  |  |  | 0.77 (0.69-0.87) | 0.80 (0.73-0.89) | 0.00 | - | - | - | - | - |  |
| Group4 |  |  |  |  |  |  | 0.42 (0.21-0.85) | 0.94 (0.66-1.35) | 0.01 | - | - | - | - | - |  |
| Age at diagnosis (year) | 1.08 (1.07-1.08) | 1.08 (1.07-1.08) | 0.01 | 1.06 (1.06-1.07) | 1.06 (1.06-1.06) | 0.00 | 1.07 (1.06-1.07) | 1.07 (1.06-1.07) | 0.01 | 1.07 (1.06-1.08) | 1.07 (1.06-1.07) | 0.01 | 1.07 (1.05-1.09) | 1.06 (1.05-1.08) | 0.01 |
| Female | 2.55 (2.19-2.98) | 2.24 (2.02-2.50) | 0.01 | 2.08 (1.92-2.26) | 1.64 (1.54-1.74) | 0.02 | - | - | - | - | - | - | - | - |  |
| Calendar year at diagnosis |  |  |  |  |  |  |  |  |  |  |  |  |  |  |  |
| 2008~2010 | 1 | 1 |  | 1 | 1 |  | 1 | 1 |  | 1 | 1 |  | 1 | 1 |  |
| 2011~2013 | 0.86 (0.78-0.94) | 0.90 (0.85-0.96) | 0.00 | 0.90 (0.82-0.98) | 1.01 (0.94-1.08) | 0.01 | 0.84 (0.76-0.93) | 0.86 (0.79-0.94) | 0.00 | 0.87 (0.75-1.01) | 0.90 (0.81-1.00) | 0.00 | 0.68 (0.41-1.11) | 0.87 (0.59-1.30) | 0.00 |
| 2014~2016 | 0.69 (0.57-0.82) | 0.75 (0.66-0.86) | 0.00 | 0.78 (0.67-0.91) | 0.87 (0.77-0.97) | 0.01 | 0.61 (0.50-0.73) | 0.68 (0.59-0.80) | 0.05 | 0.81 (0.64-1.03) | 0.99 (0.84-1.18) | 0.01 | 0.69 (0.34-1.41) | 0.71 (0.38-1.33) | 0.01 |
| Urban (vs. Rural) | 0.95 (0.87-1.03) | 0.94 (0.88-1.00) | 0.00 | 0.98 (0.90-1.05) | 0.97 (0.92-1.03) | 0.00 | 0.91 (0.82-1.01) | 0.92 (0.85-1.00) | 0.00 | 1.05 (0.91-1.20) | 0.90 (0.82-0.99) | 0.01 | 1.15 (0.74-1.79) | 1.12 (0.79-1.60) | 0.00 |
| Insurance level |  |  |  |  |  |  |  |  |  |  |  |  |  |  |  |
| 1~2 | 1 | 1 |  | 1 | 1 |  | 1 | 1 |  | 1 | 1 |  | 1 | 1 |  |
| 3~4 | 1.12 (0.96-1.31) | 0.92 (0.82-1.03) | 0.01 | 0.90 (0.77-1.06) | 0.90 (0.80-1.00) | 0.00 | 1.04 (0.91-1.18) | 1.00 (0.85-1.13) | 0.00 | 1.06 (0.79-1.42) | 1.02 (0.82-1.25) | 0.00 | 0.97 (0.50-1.88) | 0.97 (0.58-1.61) | 0.00 |
| 5~6 | 1.02 (0.88-1.19) | 0.99 (0.89-1.10) | 0.00 | 1.07 (0.92-1.23) | 0.94 (0.85-1.04) | 0.01 | 1.03 (0.90-1.19) | 0.94 (0.81-1.08) | 0.01 | 1.22 (0.93-1.59) | 1.10 (0.91-1.33) | 0.01 | 0.87 (0.46-1.67) | 0.91 (0.55-1.51) | 0.00 |
| 7~8 | 1.01 (0.88-1.16) | 0.94 (0.86-1.04) | 0.00 | 0.97 (0.85-1.11) | 0.90 (0.82-0.99) | 0.00 | 0.96 (0.82-1.12) | 0.91 (0.80-1.04) | 0.01 | 0.97 (0.76-1.25) | 1.04 (0.88-1.24) | 0.00 | 0.76 (0.40-1.46) | 0.68 (0.41-1.14) | 0.00 |
| 9~10 | 0.95 (0.84-1.07) | 0.85 (0.78-0.94) | 0.01 | 0.89 (0.79-1.01) | 0.87 (0.80-0.95) | 0.00 | 0.94 (0.81-1.09) | 1.00 (0.89-1.12) | 0.00 | 0.94 (0.75-1.17) | 1.06 (0.91-1.24) | 0.01 | 0.85 (0.48-1.52) | 0.68 (0.41-1.12) | 0.01 |
| 0 | 0.99 (0.81-1.22) | 0.94 (0.81-1.08) | 0.00 | 1.08 (0.92-1.28) | 0.98 (0.86-1.11) | 0.00 | 0.94 (0.77-1.14) | 0.99 (0.88-1.19) | 0.00 | 1.37 (1.03-1.82) | 1.30 (1.05-1.60) | 0.00 | 0.90 (0.43-1.90) | 0.94 (0.47-1.87) | 0.00 |
| SEER stage - Regional | 1.05 (0.96-1.15) | 1.04 (0.98-1.11) | 0.00 | 1.25 (1.14-1.37) | 1.26 (1.18-1.35) | 0.00 | 1.17 (1.07-1.29) | 1.20 (1.11-1.29) | 0.00 | 1.02 (0.88-1.17) | 0.97 (0.88-1.07) | 0.01 | 1.26 (0.85-1.86) | 1.38 (0.99-1.92) | 0.01 |
| CCI index |  |  |  |  |  |  |  |  |  |  |  |  |  |  |  |
| 0-1 | 1 | 1 |  | 1 | 1 |  | 1 | 1 |  | 1 | 1 |  | 1 | 1 |  |
| 2 | 1.09 (0.91-1.31) | 1.06 (0.87-1.28) | 0.00 | 1.02 (0.82-1.26) | 0.84 (0.70-1.11) | 0.01 | 1.20 (1.01-1.43) | 1.07 (0.89-1.27) | 0.01 | 0.96 (0.59-1.55) | 1.00 (0.63-1.57) | 0.00 | 0.92 (0.43-1.96) | 0.80 (0.36-1.80) | 0.00 |
| 3 | 1.28 (1.07-1.53) | 1.27 (1.07-1.52) | 0.00 | 1.24 (1.01-1.52) | 0.97 (0.82-1.15) | 0.01 | 1.46 (1.23-1.73) | 1.23 (1.04-1.45) | 0.01 | 1.66 (1.08-2.54) | 1.72 (1.17-2.54) | 0.00 | 0.87 (0.40-1.90) | 1.14 (0.56-2.31) | 0.00 |
| 4+ | 1.37 (1.17-1.60) | 1.56 (1.33-1.83) | 0.00 | 1.47 (1.23-1.77) | 1.36 (1.18-1.58) | 0.00 | 1.37 (1.18-1.60) | 1.35 (1.16-1.56) | 0.00 | 2.23 (1.54-3.23) | 1.89 (1.33-2.70) | 0.01 | 1.29 (0.70-2.35) | 1.51 (0.84-2.74) | 0.00 |
| BMD test | 1.25 (1.13-1.37) | 1.25 (1.13-1.37) | 0.00 | 2.72 (2.37-3.11) | 2.31 (2.09-2.55) | 0.01 | 1.12 (1.02-1.24) | 0.97 (0.89-1.05) | 0.01 | 1.73 (1.47-2.02) | 1.45 (1.29-1.63) | 0.01 | 1.63 (1.03-2.58) | 1.45 (0.98-2.14) | 0.01 |
| Osteoporotic medicine | 1.29 (1.11-1.50) | 1.41 (1.28-1.55) | 0.00 | 1.52 (1.18-1.96) | 1.54 (1.31-1.81) | 0.00 | 1.43 (1.20-1.70) | 1.64 (1.44-1.88) | 0.01 | 0.96 (0.79-1.17) | 0.99 (0.87-1.13) | 0.01 | 2.20 (1.19-4.07) | 2.21 (1.32-3.70) | 0.01 |
| Coefficient of Determinant (R²) | 0.019 | 0.026 | 0.01 | 0.023 | 0.03 | 0.04 | 0.019 | 0.02 | 0.01 | 0.025 | 0.029 | 0.03 | 0.012 | 0.01 | 0.02 |
